# Identification and Validation of Immune Molecular Subtypes in Pancreatic Ductal Adenocarcinoma: Implications for Prognosis and Immunotherapy

**DOI:** 10.3389/fimmu.2021.690056

**Published:** 2021-07-15

**Authors:** Ruiyu Li, Yangzhige He, Hui Zhang, Jing Wang, Xiaoding Liu, Hangqi Liu, Huanwen Wu, Zhiyong Liang

**Affiliations:** ^1^ Department of Pathology, State Key Laboratory of Complex Severe and Rare Disease, Molecular Pathology Research Center, Peking Union Medical College Hospital, Chinese Academy of Medical Sciences & Peking Union Medical College, Beijing, China; ^2^ Department of Medical Research Center, State Key Laboratory of Complex Severe and Rare Diseases, Peking Union Medical College Hospital, Chinese Academy of Medical Science & Peking Union Medical College, Beijing, China

**Keywords:** pancreatic ductal adenocarcinoma, immune molecular subtypes, non-negative matrix factorization, immunotherapy, immunogenomics

## Abstract

**Background:**

Pancreatic ductal adenocarcinoma (PDAC) remains treatment refractory. Immunotherapy has achieved success in the treatment of multiple malignancies. However, the efficacy of immunotherapy in PDAC is limited by a lack of promising biomarkers. In this research, we aimed to identify robust immune molecular subtypes of PDAC to facilitate prognosis prediction and patient selection for immunotherapy.

**Methods:**

A training cohort of 149 PDAC samples from The Cancer Genome Atlas (TCGA) with mRNA expression data was analyzed. By means of non-negative matrix factorization (NMF), we virtually dissected the immune-related signals from bulk gene expression data. Detailed immunogenomic and survival analyses of the immune molecular subtypes were conducted to determine their biological and clinical relevance. Validation was performed in five independent datasets on a total of 615 samples.

**Results:**

Approximately 31% of PDAC samples (46/149) had higher immune cell infiltration, more active immune cytolytic activity, higher activation of the interferon pathway, a higher tumor mutational burden (TMB), and fewer copy number alterations (CNAs) than the other samples (all P < 0.001). This new molecular subtype was named Immune Class, which served as an independent favorable prognostic factor for overall survival (hazard ratio, 0.56; 95% confidence interval, 0.33-0.97). Immune Class in cooperation with previously reported tumor and stroma classifications had a cumulative effect on PDAC prognostic stratification. Moreover, programmed cell death-1 (PD-1) inhibitors showed potential efficacy for Immune Class (P = 0.04). The robustness of our immune molecular subtypes was further verified in the validation cohort.

**Conclusions:**

By capturing immune-related signals in the PDAC tumor microenvironment, we reveal a novel molecular subtype, Immune Class. Immune Class serves as an independent favorable prognostic factor for overall survival in PDAC patients.

## Introduction

Pancreatic ductal adenocarcinoma (PDAC) is a fatal disease with a 5-year overall survival rate of approximately 9% (1, 2). Surgical resection remains the only curative method, and evolving adjuvant chemotherapy regimens have shown limited efficacy in improving long-term outcomes (3). The emergence of immune checkpoint blockade therapies has shed light on the treatments of PDAC patients. However, according to recent clinical trials, only a minority of PDAC patients benefit from immunotherapy (4). Moreover, although various predictive biomarkers for immunotherapy have been developed for solid tumors, none have proven their efficacy in PDAC patients (5–7). Thus, it is necessary to develop new biomarkers for immunotherapy with particular emphasis on PDAC.

Recently, a series of other molecular subtype classifications based on high-throughput expression profiling data were developed for PDAC, with the aim of prognostic stratification. These classifications included a three-subtype classification [classical, quasimesenchymal (QM), and exocrine-like] based on microdissected samples (8) and a four-subtype classification [squamous, pancreatic progenitor, immunogenic, and aberrantly differentiated endocrine exocrine (ADEX)] based on bulk samples (9). However, given that these classifications were developed using different sources of samples and different techniques, their prognostic values need to be validated in more datasets (10, 11). Moreover, these classifications were based on tumor cells rather than microenvironment compartments of PDAC. The tumor microenvironment of PDAC comprises an admixture of multiple cell types within the extracellular matrix, including cancer-associated fibroblasts (CAFs) and various kinds of immune cells (12, 13). As a robust method for unsupervised class discovery, non-negative matrix factorization (NMF) has shown the capability to detect context-dependent molecular signals from these distinct compartments (14). Moffitt et al. used NMF to virtually microdissect bulk RNA sequencing data and identify tumor subtype classification (classical and basal-like) and stroma subtype classification (normal and activated) (15). Nevertheless, few molecular classifications have focused on the immune compartments of PDAC or been correlated with the treatment efficacy of immunotherapy. Thus, further research should focus on identifying immune molecular subtypes based on the virtual microdissection of immune-related signals within the tumor microenvironment to facilitate prognostic stratification and discover effective biomarkers for immunotherapy (16).

The current research applied the NMF method to virtually dissect immune-related signals from gene expression data of PDAC samples. We identified an immune molecular subtype, Immune Class, based on the tumor immune microenvironment of PDAC. Detailed immunogenomic profiling showed that Immune Class had several characteristics, including more active immune cytolytic activity, higher immune cell infiltration, higher tumor mutational burden (TMB), and lower copy number alterations (CNAs) than Nonimmune Class. Immune Class also served as an independent favorable prognostic factor for overall survival. In addition, our immune molecular subtypes might complement the current classification systems and facilitate personalized immunotherapy. Our findings provide a comprehensive understanding of the immunological landscape in PDAC and deserve further validation in PDAC patients treated with immunotherapy.

## Materials and Methods

### PDAC Datasets and Samples

**Figure 1:**
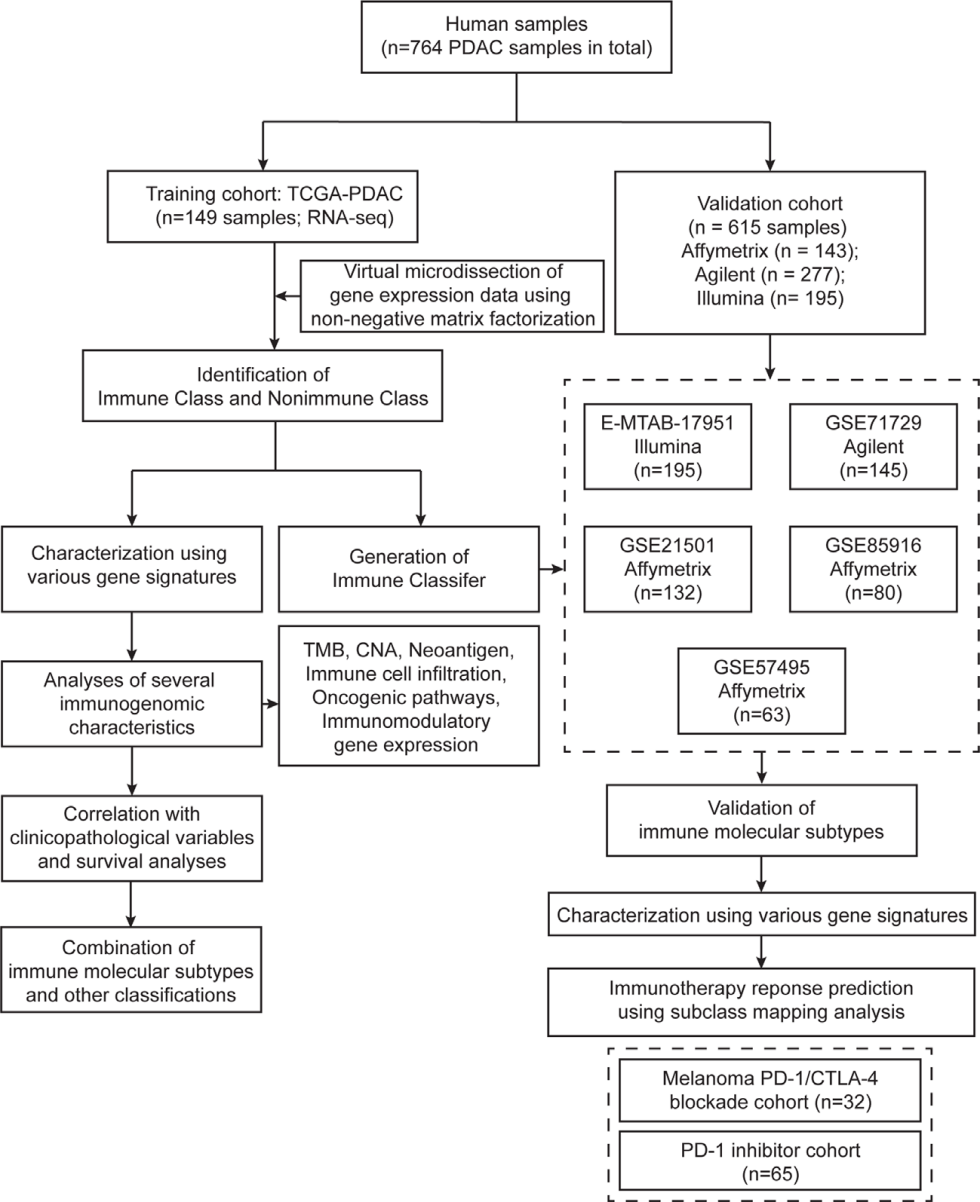
Flow chart of the study. A total of 764 PDAC samples were included in this study. A training cohort (TCGA-PDAC) including 149 samples was virtually microdissected to identify immune molecular subtypes. Detailed immunogenomic characterization was performed between the two immune molecular subtypes. The Immune Classifier was adopted in five independent validation datasets to validate the immune molecular subtypes.

We analyzed the mRNA gene expression data from a cohort of 764 patients with pancreatic cancers (**Figure 1**). A cohort of 149 PDAC samples from The Cancer Genome Atlas (TCGA) was used as the training cohort. Public level 3 HT-seq fragments per kilobase of exon model per million mapped fragments (FPKM) data were downloaded from the TCGA data port (https://portal.gdc.cancer.gov/, accessed September 16, 2020) (17). The corresponding clinicopathological information was collected at the same time, including survival time, survival status, age, sex, TNM stage, histological grade, and etc. Only primary PDAC tumor samples were included for downstream analyses. A total of 5000 genes with the highest median expression in the samples were retained for NMF analysis. Five publicly available datasets with a total of 615 samples were further used for validation (series: GSE85916, GSE71729, GSE57495, GSE21501, and E-MTAB-17951). In these datasets, gene expression was profiled using different microarray platforms ([HG-U219] Affymetrix Human Genome U219 Array, Rosetta/Merck Human RSTA Custom Affymetrix 2.0 microarray, Agilent-014850 Whole Human Genome Microarray 4x44K G4112F, and Illumina human WG6 BeadChip v3). The gene expression data were downloaded from the Gene Expression Omnibus (GEO) (https://www.ncbi.nlm.nih.gov/geo/) and Array Express (www.ebi.ac.uk/arraryexpress). The probe IDs were transformed to gene symbols with GEO platform files, and probes mapping to the same gene symbol were collapsed by mean expression. Samples were normalized using to each other using quantile normalization with the R package “*limma*” (18). The key information of these five datasets is summarized in **Supplementary Table 1**.

### Virtual Dissection of Immune-Related Gene Expression Signals and Unsupervised Class Discovery

**Figure 2:**
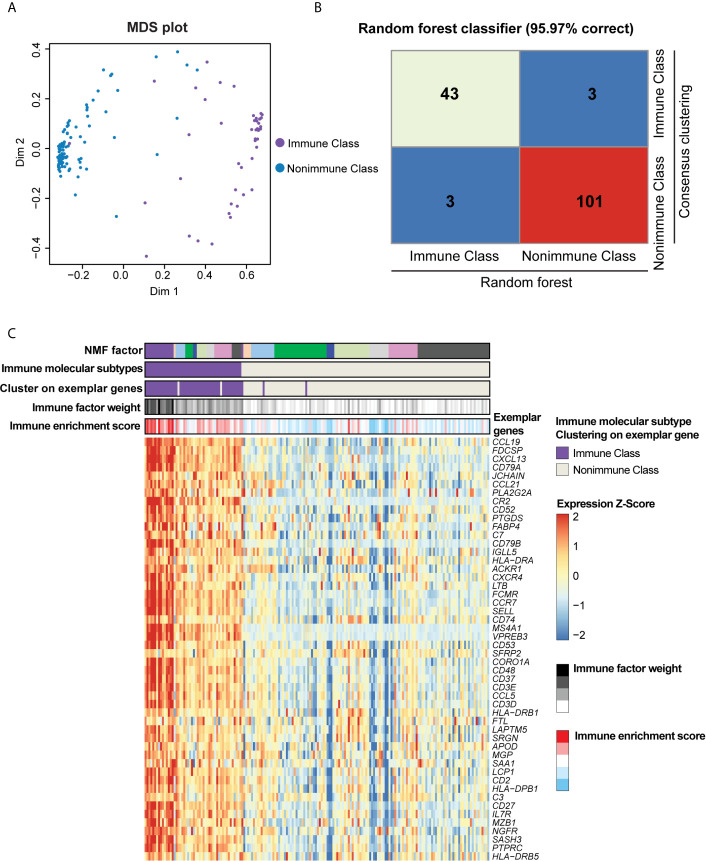
Immune molecular subtypes determined after consensus clustering and random forest refinement. **(A)** Consensus clustering of the TCGA-PDAC cohort using the exemplar genes was further refined using random forest as illustrated in the multidimensional scaling (MDS) plot. Purple dots indicated patients classified as Immune Class according to consensus clustering, and blue dots indicates patients classified as Nonimmune Class. **(B)** Heatmap of confusion matrix exhibited the correction rate of random forest classifier compared with consensus clustering. **(C)** Heatmap displayed the overlap between NMF factors, immune factor weight, immune enrichment score, consensus clustering using exemplar genes, and immune molecular subtypes. The expression of exemplar genes was illustrated at the bottom heatmap.

**Figure 3:**
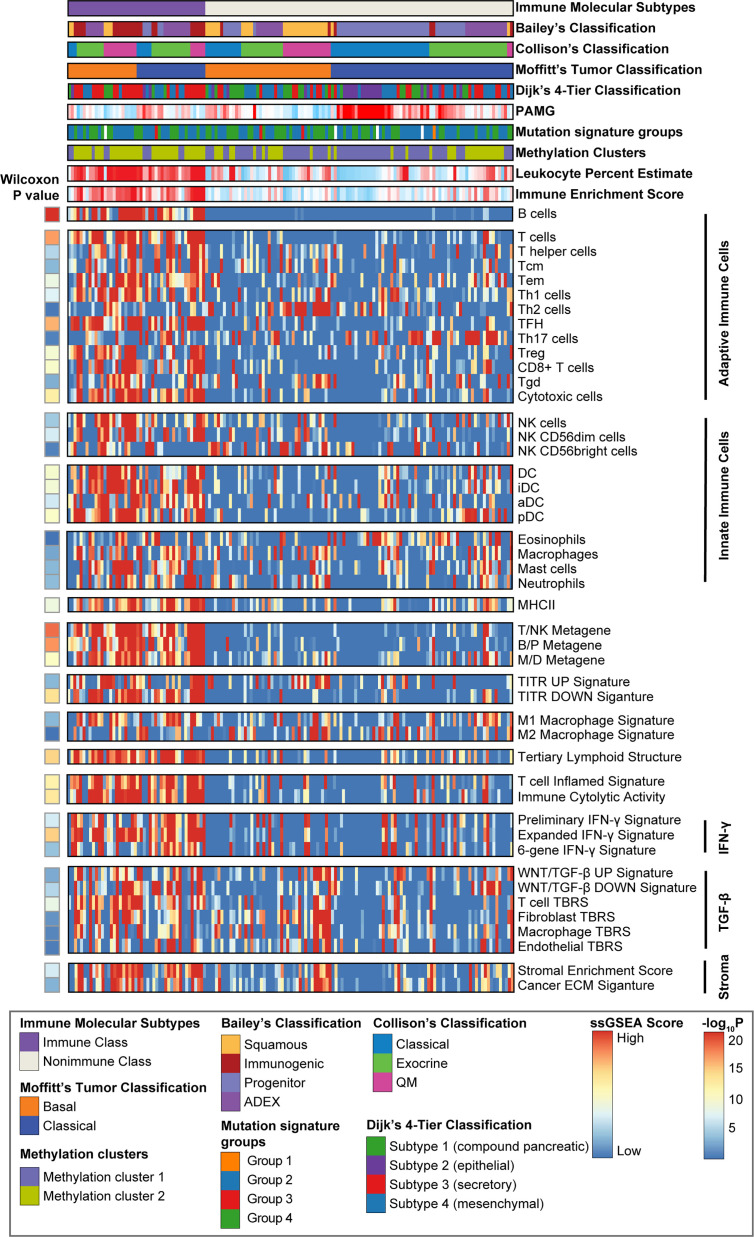
Identification of PDAC immune molecular subtypes. Consensus-clustered heatmap of the TCGA-PDAC cohort using exemplar genes of the immune non-negative matrix factorization (NMF) factor. Immune Class was indicated in purple and constituted 30.8% (46/149) of the TCGA-PDAC cohort. Single sample gene set enrichment analysis (ssGSEA) was performed using a series of gene sets, including signatures of innate and adoptive immune response. The enrichment score of ssGSEA was displayed in the heatmap. Wilcoxon rank sum test compared ssGSEA enrichment scores of the immune-related signatures between Immune Class and Nonimmune Class. Methylation estimated leukocyte percent, Bailey’s classification, Collison’s classification, and Moffitt’s tumor classification, Dijk’s 4-tier classification, pancreatic adenocarcinoma molecular gradient (PAMG), mutation signature groups and methylation clusters were also shown at the top panel. Tcm cells, central memory T cells; Tem cells, effector memory T cells; TFH cells, T follicular helper cells; Th17 cells, T helper 17 cells; Treg cells, Regulatory T cells; Tgd cells, γδ T cells; NK cells, natural killer cells; DC, dendritic cells; iDC, immature dendritic cells; aDC, activated dendritic cells; pDC, plasmacytoid dendritic cells; T/NK metagene, T cell/NK cell metagene; B/P metagene, B cell/plasma cell metagene; M/D metagene, monocyte/dendritic cell metagene; TITR, tumor infiltrating regulatory T cells; IFN-γ, interferon-γ; TGF-β, transforming growth factor-β; TBRS, TGF-β response signature; ECM, extracellular matrix.

The tumor, stromal, and immune cell gene expression signals in the training TCGA-PDAC cohort were deconvoluted and virtually microdissected using NMF as previously described (14, 15) with the R package “*NMF*” (19). k = 9 was selected as the number of factorization factors because it could achieve high cophenetic coefficients and provide effective deconvolution of the TCGA-PDAC cohort in terms of immune-related signals. The coefficient matrix and basis matrix are displayed in **Supplementary Table 2**. We applied a previously reported immune enrichment score calculated by single-sample gene set enrichment analysis (ssGSEA) (20) to obtain the immune-related NMF factor. The nine NMF factors were compared to the immune enrichment score, and the NMF factor with the highest level of immune enrichment score was subsequently referred to as the immune factor (**Supplementary Figure 1A**). The top-ranked genes by their loadings of the immune factor are herein referred to as exemplar genes (**Supplementary Table 3**). The top 100 exemplar genes of the immune factor were subjected to Gene Ontology (GO) enrichment and Kyoto Encyclopedia of Genes and Genomes (KEGG) pathway analyses (21–23). A false discovery rate (FDR)-adjusted P-value < 0.05 was considered as the criterion for significant enrichment for GO terms and KEGG pathways. Subsequently, the top 50 exemplar genes of the immune factor were selected for unsupervised consensus clustering to divide the TCGA-PDAC cohort into two immune molecular subtypes: Immune Class and Nonimmune Class (**Figures 2C**, **3**). Finally, the subtypes obtained from consensus clustering were further refined with the R package “*Random Forest*” (24); thus, the final Immune Class and Nonimmune Class were identified. The multidimensional scaling (MDS) plot and confusion matrix are displayed in **Figures 2A, B**.

### Molecular Characteristics of the Immune Molecular Subtypes and the Generation of Immune Molecular Subtype Classifiers

Sets of previously reported immune- and stroma-related gene expression signatures representing immune cell infiltration and immune responses are summarized in **Supplementary Table 4**. We applied these gene sets to characterize the immune molecular subtypes using ssGSEA and nearest template prediction (NTP). ssGSEA was conducted using the R package “*GSVA*” (25), and NTP was conducted using an R version of the GenePattern module NTP (26, 27). The Estimation of Stromal and Immune cells in MAlignant Tumor tissues using Expression data (ESTIMATE) algorithm was used to calculate the immune enrichment score and the stromal enrichment score with the R package “*ESTIMATE*” (20). Differential analyses between the two immune molecular subtypes revealed differentially expressed genes (DEGs). Linear models were used to identify DEGs with the R package “*limma*” (18). An FDR < 0.05 combined with |log2(fold change)| ≥ 1.5 was set as the threshold for DEG identification. The DEGs reaching the threshold were considered the Immune Classifier. Genes whose expression was higher in Immune Class than in Nonimmune Class were considered as classifier genes of Immune Class, and vice versa. Gene set enrichment analysis (GSEA) was utilized to identify differential enrichment of the immune-related pathways, infiltrating immune cells, and immune responses (28). GSEA was performed using GSEA software version 4.1.0 from the Broad Institute, and gene sets (as gene symbols version 7.2) were downloaded from the Molecular Signatures Database (http://software.broadinstitute.org/gsea/msigdb). The normalized enrichment score (NES) was obtained by 1000 permutations. Gene sets with a p-value < 0.05 and an FDR < 0.25 were considered significantly enriched. All heatmaps were generated using the R package “*pheatmap*”.

### Correlations of the Immune Molecular Subtypes With Immunogenomic Features

CNA data generated by GISTIC2.0 were obtained from the Broad Institute GDAC FireBrower (http://firebrowse.org). Arm-level amplifications and deletions were defined by gains or loss in each chromosome. The numbers of both arm- and focal-level CNAs were compared between Immune Class and Nonimmune Class using the Wilcoxon rank-sum test. The mutation data of the TCGA-PDAC cohort were downloaded from the TCGA data portal (https://tcga-data.nci.nih.gov/tcga), and TMB was calculated as the number of nonsynonymous mutations per million bases. We used the MutSig2.0 approach (29) to identify and visualize significantly mutated genes (SMGs) with the R package “*Maftools*” (30), and mutations in known driver genes of PDAC and genes in the WNT/β-catenin pathway were visualized in an oncoplot. Illumina Infinium human methylation 450K array data was downloaded from UCSC Xena (http://xena.ucsc.edu/). Pathological tumor cellularity, ABSOLUTE purity, DNA hypermethylation purity, and the DNA methylation-estimated leukocyte fraction were obtained from a previous study on the genomic characterization of PDAC (31). Twenty-two subpopulations of tumor-infiltrating lymphocytes (TILs) were analyzed using the CIBERSORT algorithm (https://cibersort.stanford.edu/) in R (32). A list of immunomodulatory genes was obtained from a previous publication (33), and the mRNA expression profiles of these genes were compared between Immune Class and Nonimmune Class.

### Combination of the Immune Molecular Subtypes and Other PDAC Molecular-Subtype Classifications

The correlations between our immune molecular subtypes (Immune Class and Nonimmune Class) and previously reported PDAC molecular subtypes were analyzed. Bailey et al. reported a four-subtype classification of pancreatic cancer: squamous, progenitor, immunogenic, and ADEX (9). Collison et al. reported a three-subtype classification: QM, classical, and exocrine-like (8). Moffitt et al. proposed two classifications of pancreatic cancer through virtual microdissection of the tumor epithelium and stromal components in the tumor microenvironment (15). The tumor classification contained the classical and basal subtypes, whereas the stromal classification contained the activated and normal subtypes. We defined these four molecular subtype classifications in each sample from the TCGA-PDAC cohort using the published classifier genes. The distribution of the aforementioned classifications in immune molecular subtypes was compared using Fisher’s exact test. A Sankey diagram was generated using the R package “*networkD3*”. Cramer’s V statistic was applied to measure the similarity between two categorical variates, herein different PDAC molecular subtype classifications. A Venn diagram comparing the classifier genes of different classifications was plotted using the R package “*VennDiagram*”. The association between clinicopathologic characteristics and overall survival in the TCGA-PDAC cohort was analyzed using uni- and multivariate Cox proportional hazards (CoxPH) regression models. Kaplan-Meier survival analysis was employed to visualize the overall survival, and the log-rank test was used to compare differences among different curves. A forest plot was plotted using the R package “*forest plot*”. PDAC molecular classifications in some recent studies were also reproduced in the TCGA-PDAC cohort. Hierarchal clustering was performed using the function “hclust” in R. Pancreatic adenocarcinoma molecular gradient was generated using the R package “*pdacmolgrad*”. Consensus clustering was performed using the R package “*ConsensusClusterPlus*”. Mutation signatures were downloaded from COSMIC (https://cancer.sanger.ac.uk/signatures/) and identified using the R package “*deconstructSigs*”.

### Validation of the Immune Molecular Subtypes in Independent External Datasets

The expression of a customized 795-gene NanoString panel in 32 patients receiving sequential cytotoxic T lymphocyte-associated antigen-4 (CTLA-4) inhibitors and programmed cell death 1 (PD-1) inhibitors was profiled in a previous study (34). Subclass mapping was performed *via* a bioinformatic approach to identify common subtypes between independent cohorts (35). The similarity of the expression of these genes between patients in the TCGA-PDAC cohort and immune checkpoint blockade responders was evaluated using subclass mapping in the GenePattern SubMap module. The Immune Classifier genes were used to predict immune molecular subtypes in five independent external validation datasets using NTP. Immune-related gene signatures (**Supplementary Table 4**) further validated and characterized the presence of immune molecular subtypes in these validation datasets. Treatment response to immunotherapy was also predicted in the validation datasets using SubMap.

### Statistical Analysis

All statistical analyses were conducted in R software (version 4.0.1) (http://www.r-project.org). Correlations between continuous variables and immune molecular subtypes were analyzed using Student’s t-test and the Wilcoxon rank-sum test for normally distributed and nonnormally distributed data, respectively. Correlations between categorical variates and immune molecular subtypes were analyzed using the chi-square test or Fisher’s exact test. Survival analysis, including CoxPH regression, Kaplan-Meier survival analysis and log-rank tests, was performed using the R packages “*survival*” and “*survminer*”. A two-sided P value < 0.05 was considered statistically significant.

## Results

### Identification of the Immune Molecular Subtypes Through Virtual Microdissection

With the aim of virtually microdissecting immune-related signals from bulk gene expression data, we performed unsupervised NMF analysis of 149 PDAC samples in the TCGA cohort (training cohort, **Figure 1**). Among the different expression patterns determined by NMF, one was correlated with a previously reported immune enrichment score reflecting the presence of infiltrating immune cells in tumor tissues (**Supplementary Figure 1A**) (20). Thus, this expression pattern was regarded as the immune NMF factor, and the top-ranked genes with the highest weight contributing to the immune NMF factor were considered as exemplar genes. Enrichment analyses of GO terms and KEGG pathways on exemplar genes provided additional evidence of immune-related functions and signaling (**Supplementary Figures 1B, C** and **Supplementary Table 5**). For example, enriched biological processes included the response to interferon-gamma (IFN-γ) and positive regulation of T cell activation. By utilizing consensus clustering on exemplar genes and random forest refinement (**Figures 2A, B**), immune molecular subtypes were identified and further referred to as “Immune Class” and “Nonimmune Class” (**Figure 2C**). Immune Class accounted for 30.8% (46/149) of the training cohort and exhibited higher expression of exemplar genes and higher immune enrichment score than Nonimmune Class (**Figures 2**, **3**).

ssGSEA revealed significant enrichment of a series of gene sets associated with innate and adaptive immune cell subpopulations, including B cells, cytotoxic T cells, and natural killer cells (NK cells), in Immune Class (all P < 0.0001) (**Figure 3**). Significant enrichment of tumor-suppressing Th1 cells, not tumor-promoting Th2 cells (P = 0.38), was also observed in Immune Class (P = 1.5e-07). Similarly, we found enrichment of a proinflammatory M1 macrophage signature (P = 6.3e-04) rather than an anti-inflammatory M2 macrophage signature (P = 0.61) in Immune Class. A six-gene IFN-γ signature that was reported to induce programmed death ligand 1 (PD-L1) expression and predict the therapeutic efficacy of the PD-1 inhibitor pembrolizumab in head and neck squamous cell carcinoma (34) was also significantly enriched in Immune Class (P = 1.6e-04). Other signatures significantly enriched in Immune Class included tertiary lymphoid structure, immune cytolytic activity, WNT/β-catenin and transforming growth factor (TGF-β) pathway, and stromal enrichment score (all P < 0.0001).

Class comparison analysis revealed 95 genes that were significantly overexpressed in Immune Class and 5 genes that were significantly overexpressed in Nonimmune Class (**Supplementary Table 6**). The Immune Classifier was further built based on the expression of this set of 100 genes (**Supplementary Table 7**). The Immune Classifier was mainly composed of immune-related genes, for example, B cell surface markers such as *CD19*, membrane spanning 4-domains A1 (*MS4A1*, CD20), *CD79A* and *CD79B*, and T cell surface markers such as *CD2*, *CD3D*, *CD3E*, and *CD5*. Several immunoglobulin genes were also overexpressed in Immune Class and included in the Immune Classifier, such as the Fc fragment of IgE receptor II (*FCER2*), Fc receptor-like 1/2/3 (*FCRL1/2/3*) and joining chain of multimeric IgA and IgM (*JCHAIN*). Furthermore, chemokine receptor and ligand genes, such as C-X-C motif chemokine ligand 12/13 (*CXCL12/13*), C-C motif chemokine ligand 19/21 (*CCL19/21*), and C-C motif chemokine receptor 4/7 (*CCR4/7*) were presented in the Immune Classifier. Granzyme genes (granzyme K/E, *GZMK* and *GZME*) were also overexpressed in Immune Class, indicating the cytotoxic activity of T cells and NK cells. Similarly, GSEA was employed to analyze the enrichment of immune cells, IFN-α and IFN-γ responses, tumor necrosis factor-α (TNF-α) signaling, Janus kinase/signal transducer and activator of transcription (JAK/STAT) signaling, and WNT/β-catenin signaling (all p < 0.05 and FDR < 0.25, **Supplementary Figure 2** and **Supplementary Table 8**).

In conclusion, by performing virtual microdissection in PDAC, we identified an immune molecular subtype named Immune Class and demonstrated the potential of Immune Class to capture signatures of immune cell infiltration, innate and adaptive immune responses, and immune-related pathways such as interferon signaling and WNT/β-catenin signaling.

### Correlations Between the Immune Molecular Subtypes and Immunogenomic Characteristics

**Figure 4:**
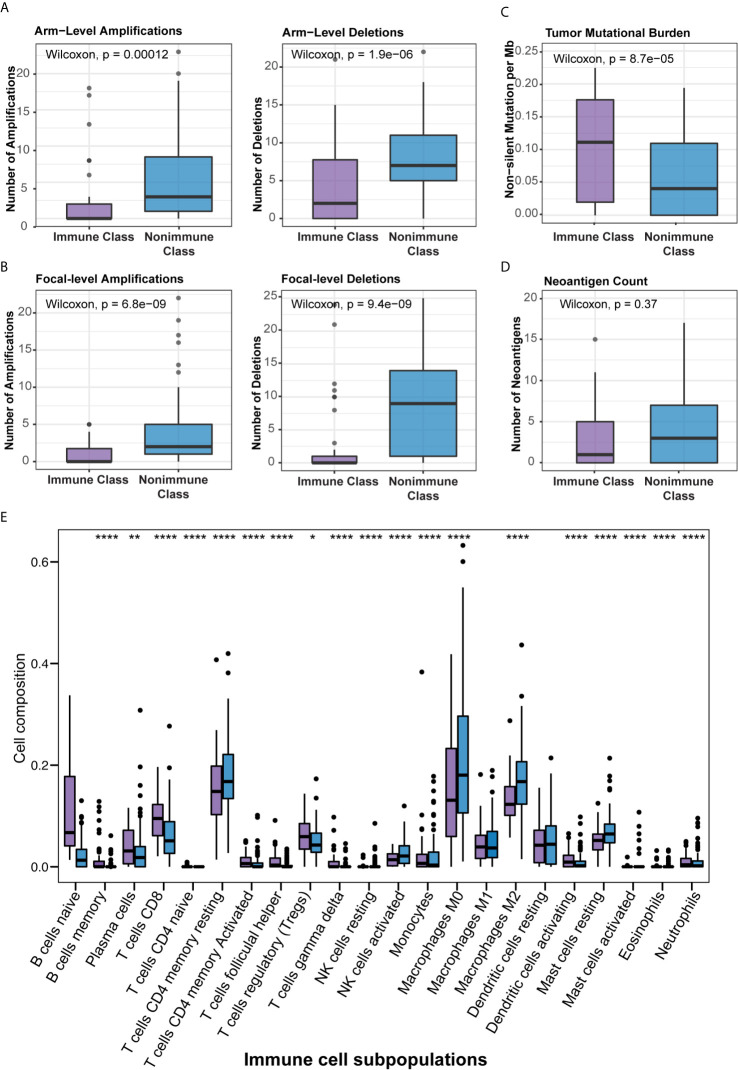
Correlation of the immune molecular subtypes with immunogenomic characteristics and immune cell infiltration. **(A, B)** Patients within Immune Class showed significantly fewer both arm-level **(A)** or focal-level **(B)** amplifications and deletions compared with patients within Nonimmune Class. **(C)** Patients within Immune Class showed significantly higher tumor mutational burden (TMB) compared with patients within Nonimmune Class. **(D)** Neoantigen count did not differ between Immune Class and Nonimmune Class. **(E)** The relative proportions of 22 immune cell subpopulations estimated by CIBERSORT were compared between the immune molecular subtypes. *p ≤ 0.05; **p ≤ 0.01; ****p ≤ 0.0001.

Several previous studies have correlated certain immunogenomic characteristics with immune cell infiltration and the antitumor immune response. In a recent study, the number of major histocompatibility complex (MHC) Class I-associated neoantigens and driver gene mutations reflected the cytolytic activity of local immune infiltration (35). In particular, a higher neoantigen load and more abundant CD8+ T cell infiltration stratified pancreatic cancer patients who survived longer survival and might guide the application of immunotherapies (36). It was also demonstrated that TMB and CNAs were associated with CD8+ T cell infiltration and immune cytolytic activity and served as independent predictive factors for the immune checkpoint blockade response (35, 37, 38). To further demonstrate the biological relevance of Immune Class, we carried out detailed immunogenomic profiling including CNAs, TMB, tumor neoantigens, TILs, etc. Immune cytolytic activity was higher in Immune Class than in Nonimmune Class (P = 4.2e-13), as were the immune enrichment score (P = 1.8e-14) and the methylation-estimated leukocyte fraction (P = 9.3e-14) (**Figure 3**). For CNAs, it is worth noting that patients classified as Immune Class had relatively fewer both arm-level amplifications and deletions. In particular, Immune Class had a median of 0 arm-level amplifications (range 0-18) and 2 arm-level deletions (range 0-21) versus a median of 3 arm-level amplifications (range 0-23) and 7 arm-level deletions (range 0-22) in Nonimmune Class (P = 0.00012, and P = 1.9e-06 respectively, **Figure 4A**). It was also demonstrated that Immune Class harbored a median of 0 focal amplifications (range 0-5) and a median of 0 focal deletions (range 0 to 24), both of which were lower in Immune Class than in Nonimmune Class with a median of 0 focal amplifications (range 0-22) and a median of 9 focal deletions (range 0-25) (P = 6.8e-09, and P = 9.4e-09 respectively, **Figure 4B**). Notably, TMB (P = 8.7e-05, **Figure 4C**) but not neoantigen count (P = 0.37, **Figure 4D**) was higher in Immune Class than in Nonimmune Class. These results demonstrate that patients within Immune Class have several immunogenomic characteristics, such as higher immune cytolytic activity, a higher leukocyte fraction, a higher TMB, and fewer arm- and focal-level CNAs than patients within Nonimmune Class.

**Figure 5:**
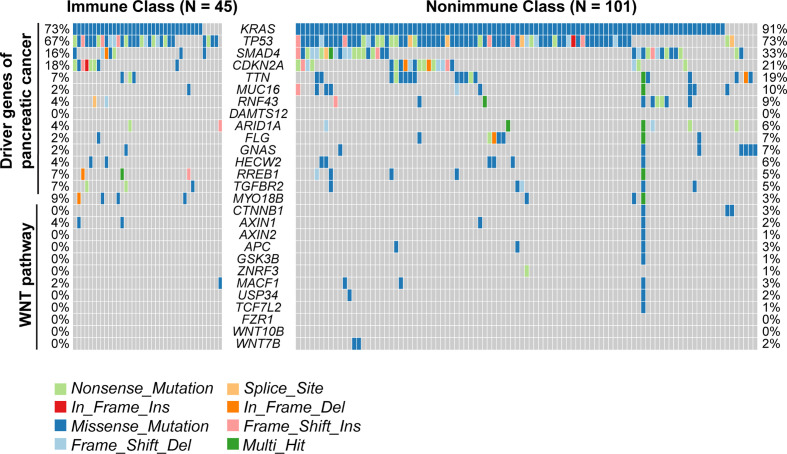
Mutations of the immune molecular subtypes. The distribution of mutations in known driver genes of PDAC and genes in the WNT/β-catenin pathway across 149 TCGA-PDAC samples were visualized in the oncoplot, including somatic nonsynonymous mutations (missense, nonsense, frame shift insertion, frame shift deletion, In-frame insertion, In-frame deletion, and splice site mutation). The mutation rates of relative genes were displayed and compared between Immune Class and Nonimmune Class.

We next sought to correlate the immune molecular subtypes with TIL subpopulations, immunomodulatory gene expression, and mutations in known driver genes of PDAC and genes in the WNT/β-catenin pathway. After deploying the CIBERSORT approach, macrophages accounted for the highest proportion of infiltrating immune cells in PDAC (**Supplementary Figure 3B**). Immune Class exhibited higher infiltration of memory B cells, CD8^+^ T cells, γδ T cells, activating NK cells, and activating dendritic cells (all P < 0.001, **Figure 4E**), which are critical in the adaptive and innate immune responses. In contrast, Nonimmune Class was enriched in M0 and M2 macrophages, yet no significant difference in M1 macrophages was observed. Moreover, we analyzed the expression of immunomodulatory genes, including both immunostimulatory and immunoinhibitory molecules that were critical for immunotherapy by supporting the immune response (39). The expression of immunomodulatory genes varied between immune molecular subtypes (**Supplementary Figure 3A**). The expression of immune checkpoint molecules such as *CTLA4* and *CD274* (PD-L1), as well as *IFNG* in IFN-γ signaling, was higher in Immune Class. Furthermore, the immune molecular subtypes were correlated with mutations in known driver genes of PDAC and genes in the WNT/β-catenin pathway (**Figure 5**). Immune Class had significantly fewer mutations in WNT/β-catenin pathway genes than Nonimmune Class (3/(45×12) versus 19/(101×12), P = 0.079). Additionally, there were significantly fewer mutations in *SMAD4* in Immune Class than in Nonimmune Class (7/45 versus 33/101, P = 0.032). Nevertheless, there was no difference in the mutation rates between Immune Class and Nonimmune Class in terms of other PDAC driver genes, such as *KRAS*, *TP53*, and *CDKN2A*. Altogether, these findings imply that immune molecular subtypes showed differences in TIL subpopulations, immunomodulatory gene expression, and certain oncogenic pathway mutations.

### Correlations of the Immune Molecular Subtypes With Clinicopathological Characteristics and Survival Analyses

**Figure 6:**
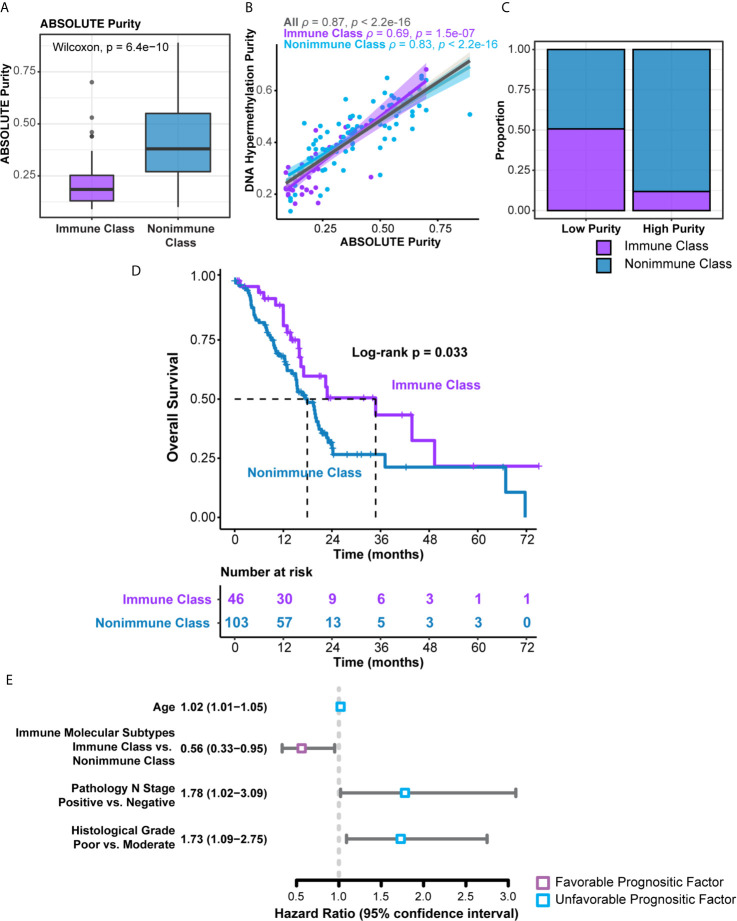
Distribution of tumor purity and survival analyses in the immune molecular subtypes. **(A)** Patients within Immune Class showed significantly lower ABSOLUTE purity compared with patients within Nonimmune Class. **(B)** ABSOLUTE purity and DNA methylation estimated purity showed strong correlation. Spearman’s ρ values were evaluated independently in Immune Class and Nonimmune Class. **(C)** Proportions of Immune Class and Non-Immune Class were compared in low and high tumor purity class. **(D)** Kaplan-Meier curves of overall survival were plotted according to the immune molecular subtypes in the TCGA-PDAC cohort. **(E)** Forest plot displayed the hazard ratio and 95% confidence interval of immune molecular subtypes and several clinicopathological characteristics for overall survival.

The clinicopathological characteristics of the TCGA-PDAC cohort were summarized and compared between Immune Class and Nonimmune Class (**Supplementary Table 9**). Immune molecular subtypes were not associated with most clinicopathological characteristics, including age, gender, lymph node invasion, and distant metastasis. Nonetheless, Immune Class was more likely to be classified as stage T1/T2 (7/46 versus 14/103) and less likely to be classified as stage T3 (35/46 versus 89/103) (P = 0.035). Tumor purity was correlated with immune and stromal cell infiltration as well as immune cytolytic activity. Tumor purity could also confound the interpretation of genomic profiling and classifications based on bulk tumor samples (40, 41). Low tumor purity was associated with Bailey’s ADEX and immunogenic subtypes and might also serve as a prognostic factor (31, 42, 43). Thus, we collected pathologist-reviewed tumor cellularity data and adopted different tumor purity estimation methods *in silico* (**Supplementary Table 10**). ABSOLUTE purity, evaluated by the whole-exome sequencing algorithm, ranged from 9.0% to 89.0% (median, 33.5%) in the whole cohort. The ABSOLUTE purity of Immune Class [median (range), 18.5% (9.0%-70.0%) was significantly lower than that of Nonimmune Class [median (range), 38% (10%-89%)] (**Figure 6A**, P = 6.4e-10) (44). Tumor purity was also estimated using DNA methylation profiles and ranged from 13.5% to 68.1% (median, 40.2%) in the whole cohort and was strongly correlated with ABSOLUTE purity (Spearman’s ρ = 0.87, p < 1e-15, **Figure 6B**) (31). A binary purity classification based on regional copy number burden indicated that Immune Class was more likely to be classified as low purity (**Figure 6C**, P < 0.001) (31). In summary, Immune Class had a lower tumor grade and lower tumor purity than Nonimmune Class.

We next sought to explore the prognostic values of the immune molecular subtypes along with other clinicopathological characteristics (**Table 1**). In univariable Cox regression analyses, immune molecular subtypes, together with age, lymph node invasion status, and histological grade, were significantly associated with overall survival. Immune Class was a favorable prognostic factor, with a hazard ratio (HR) of 0.56 [95% confidence interval (95% CI) 0.33-0.95, P = 0.033]. The median survival time of Immune Class was 34.8 months (95% CI = 16.4-not reached), which was longer than that of Nonimmune Class (17.9 months, 95% CI = 15.1-21.4). Kaplan-Meier curves also showed that Immune Class was associated with better overall survival (**Figure 6D**). Moreover, the HR of age was 1.02 (95% CI = 1.01-1.05, P = 0.036), and the HR of lymph node invasion was 1.78 (95% CI = 1.02-3.09, P = 0.008). In addition, the HR of poor versus moderate histological grade was 1.73 (95% CI = 1.09-2.75, P = 0.02). These four prognostic factors were presented in a forest plot (**Figure 6E**) and subsequently examined using multivariable Cox regression analysis. Older age [HR (95% CI) = 1.03 (1.01-1.05), P = 0.026] remained an independent unfavorable prognostic factor, whereas Immune Class remained an independent favorable prognostic factor for overall survival [HR (95% CI) = 0.56 (0.33-0.97), P = 0.037] (**Table 1**). Additionally, various metrics of tumor purity and immune infiltration, including ABSOLUTE/methylation purity, the immune enrichment score, and the methylation-estimated leukocyte fraction, were not prognostic (**Table 1**). Our results indicated that Immune Class could serve as an independent prognostic factor in PDAC.

**Table 1:** Uni- and multivariate Cox proportional hazards regression analysis of the immune molecular subtypes and clinicopathological characteristics.

Variables	Uni-variable		Multi-variable	
	HR (95% CI for HR)	P value	HR (95% CI for HR)	P value
**Age**	1.02 (1.01-1.05)	**0.036**	1.03 (1.01-1.05)	**0.026**
**Sex**				
Female	1.00	-		
Male	0.81 (0.52-1.27)	0.360		
**Immune Molecular Subtypes**				
Nonimmune Class	1.00	-	-	-
Immune Class	0.56 (0.33-0.95)	**0.033**	0.56 (0.33-0.97)	**0.037**
**pTNM Stage**				
Stage I/II	1.00	-		
Stage III/IV	1.05 (0.33-3.35)	0.933		
**Primary Tumor (T Stage)**				
T1/T2	1.00			
T3	1.61 (0.77-3.36)	0.208		
T4	1.04 (0.13-8.35)	0.969		
**Lymph Nodes (N Stage)**				
Negative	1.00	-	1.00	-
Positive	1.78 (1.02-3.09)	**0.008**	1.65 (0.95-2.88)	0.078
**Distant Metastasis (M Stage)**				
Negative	1.00	-		
Positive	1.95 (0.47-8.14)	0.362		
Not measurable	1.01 (0.64-1.59)	0.976		
**Histological Grade**				
Moderate	1.00	-	1.00	-
Poor	1.73 (1.09-2.75)	**0.020**	1.76 (1.11- 2.8)	0.017
Others	0.57 (0.14-2.36)	0.435	0.47 (0.11-2.00)	0.311
**Primary Site**				
Head	1.00	-		
Tail or body	0.88 (0.48-1.64)	0.690		
Others	0.14 (0.02-0.99)	0.050		
**History of Chronic Pancreatitis**				
No	1.00	-		
Yes	1.04 (0.49-2.20)	0.914		
**ABSOLUTE Purity**	2.12 (0.69-6.49)	0.191		
**DNA Hypermethylation Purity**	3.60 (0.65-19.77)	0.141		
**Purity Class**				
Low	1.00	-		
High	1.34 (0.85-2.11)	0.207		
**DNA Methylation Leukocyte Fraction**	0.37 (0.07-1.92)	0.237		
**Immune Enrichment Score**	0.99 (0.99-1.00)	0.082		

95% CI, 95% confidence interval; HR, hazard ratio. Bold values denote statistical significance at the P < 0.05 level.

To further explore the relationship with other transcriptome-based PDAC classifications, we included two another PDAC classifications, pancreatic adenocarcinoma molecular gradient (PAMG) (45) and Dijk’s 4-tier classification (46). The PAMG was a summary of all previous epithelial molecular classification of PDAC, while Dijk’s 4-tier classification intend to build a unifying transcriptome-based classifications. We reproduced these two tumor epithelial classifications in the TCGA-PDAC cohort. Immune Class had lower molecular gradient compared to Nonimmune Class (t-test, P = 5.1e-5, **Figure 3**). As for Dijk’s 4-tier classification, we found that the Immune Class had a higher proportion of secretory subtypes compared to Nonimmune Class (Fisher’s exact P = 0.002, **Figure 3**). Several PDAC classifications based on genome and methylome were also built recently, including mutation signature subtypes (47), homologous recombination deficiency (HRD) (48), and methylation clusters (49). We also reproduced mutation signature subtypes and methylation clusters in the TCGA-PDAC cohort. The mutation signature subtypes used NMF and hierarchical clustering to define four major subtypes. Nevertheless, we failed to discover correlation between the immune molecular subtypes and mutation signature subtypes (Fisher’s exact P = 0.91, **Figure 3**). We observed a higher proportion of Methylation Cluster2 (Methylation^low^/IFNsignature^high^) in the Immune Class (Fisher’s exact P = 3.2e-7, **Figure 3**), which was consistent with our findings that Immune Class had higher enrichment of IFN-α and IFN-γ signaling. In conclusion, these results highlighted the potential mechanisms of DNA methylation in modulating tumor immune microenvironment. And the correlation between immune molecular subtypes and alterations in genome and methylome needs further research.

### Combination of the Immune Molecular Subtypes With PDAC Tumor and Stroma Classifications for Prognostic Stratification

**Figure 7:**
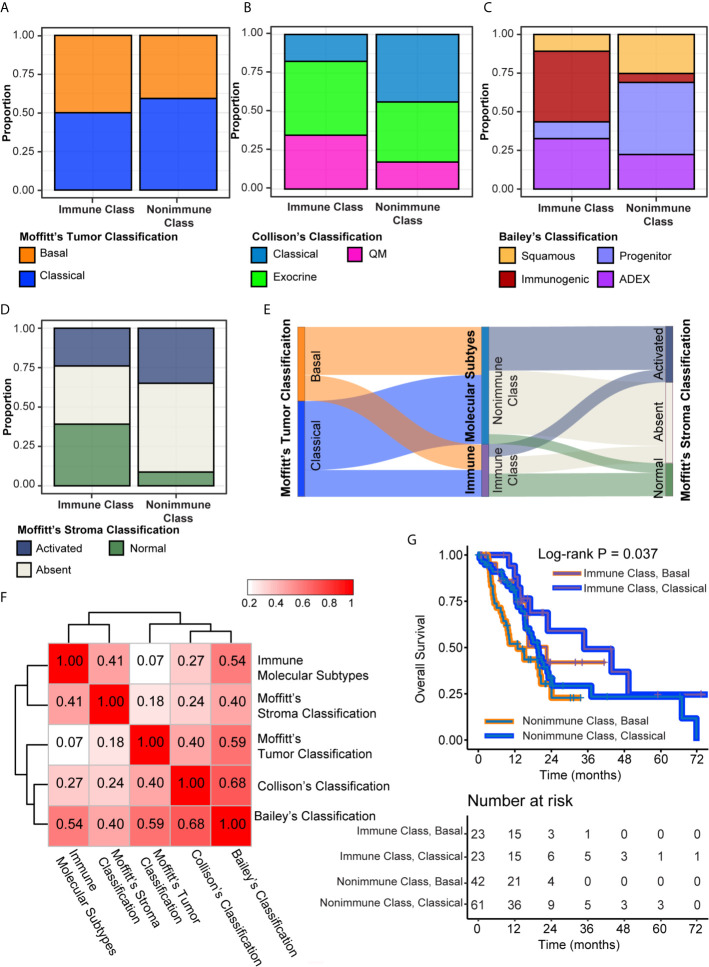
Integration of the immune molecular subtypes and other PDAC classifications. **(A–D)** Distribution of Moffitt’s tumor classification **(A)**, Collison’s classification **(B)**, Bailey’s classification **(C)**, and Moffitt’s stroma classification **(D)** were compared between Immune Class and Nonimmune Class. **(E)** Sankey chart displayed the distribution of Moffitt’s tumor classification, Moffitt’s stroma classification, and immune molecular subtypes. **(F)** Heatmap of Cramer’s V statistic reflected the corrections between five PDAC molecular classifications. **(G)** Kaplan-Meier curves of overall survival were plotted according to the immune molecular subtypes and Moffitt’s tumor classification.

Four molecular classifications of PDAC based on gene expression profiles that were biologically and clinically relevant in different sets of patients showed concordance to some extent (8, 9, 15). We evaluated the correlations of the immune molecular subtypes with these classifications and further explored the integration of immune molecular subtypes with tumor and stroma classifications in prognostic stratification. The classifier genes of Moffitt’s tumor, Moffitt’s stroma, Collison’s, and Bailey’s subtypes were used to cluster patients in the TCGA-PDAC cohort by NTP. The distributions of these four classifications were compared with the distribution of immune molecular subtypes using Fisher’s exact test (**Supplementary Table 11**). There was no significant difference between the distributions of Moffitt’s tumor subtypes and immune molecular subtypes, probably because virtual microdissection was utilized to deconvolute tumor cell signals in the study by Moffitt et al. (P = 0.38, **Figures 7A**, **F**). Nevertheless, significant correlations between immune molecular subtypes and other PDAC classifications, including Collison’s subtypes, Bailey’s subtypes and Moffitt’s stroma subtypes, were revealed (all P < 0.005). For the integration with Collison’s classification, the proportion of the classical subtype was significantly lower and the proportion of the QM subtype was significantly higher within Immune Class versus Nonimmune Class (17.4% versus 43.8%, 34.8% versus 17.5%, P = 0.004, respectively) (**Figure 7B**). For Bailey’s classification, the frequency of ADEX and immunogenic subtypes was higher within Immune Class versus Nonimmune Class (45.7% versus 5.83% and 32.6% versus 22.3%, P < 0.001, respectively) (**Figure 7C**). In contrast, we also observed a lower frequency of squamous and progenitor subtypes within Immune Class compared to Non-Immune Class (10.9% versus 25.2%, 10.9% versus 46.6%, respectively). For Moffitt’s stroma subtypes, we found that Immune Class was composed of a more normal stroma subtype and a less activated stroma subtype than Nonimmune Class (43.4% versus 8.74% and 23.9% versus 36.9%, P < 0.001, respectively) (**Figure 7D**). The Sankey diagram illustrated the PDAC assignment according to the immune molecular subtypes and Moffitt’s tumor/stroma classifications (**Figure 7E**). Analysis based on Cramer’s V statistic demonstrated a strong correlation between the immune molecular subtypes and Bailey’s classifications (Cramer’s V value = 0.54) and a weak correlation between the immune molecular subtypes and Moffitt’s tumor classifications (Cramer’s V value = 0.07). The overlap among the Immune Classifier genes, Moffitt’s tumor/stroma classifier genes is illustrated in the Venn Diagram (**Supplementary Figure 4A**). In conclusion, it was demonstrated that Immune Class was correlated with a higher proportion of the QM/ADEX subtypes, immunogenic subtype, and normal stroma subtype.

The cumulative effect of different classifications based on the tumor epithelium, stromal, and immune cells on prognostic stratification was next explored (10). In univariable Cox regression analyses, Moffitt’s stroma classification, instead of Moffitt’s tumor, Bailey’s and Collison’s classifications, had prognostic value in the TCGA-PDAC cohort (**Supplementary Table 12**). The normal stroma subtype was associated with significantly longer overall survival than the other stroma subtypes, with an HR of 0.46 (95% CI = 0.24-0.93, P = 0.03) (**Supplementary Figure 4B**). Integration of Moffitt’s tumor/stroma classification in survival analyses did not hamper the prognostic value of the immune molecular subtypes (**Figure 7G** and **Supplementary Figures 4C, D**). Patients within Immune Class and the classical tumor subtype had the longest median survival time of 34.8 months, whereas patients within Nonimmune Class and the basal tumor subtype had the shortest median survival time of 12.9 months (log-rank P = 0.009, **Figure 7G**). Since the difference between the activated stroma subtype and the absent stroma subtype was not significant for overall survival (HR [95% CI], 0.85 [0.52-1.41], P = 0.54), we combined these two stroma subtypes into other stroma subtypes and compared them with the normal stroma subtype. After integrating the immune molecular subtypes and Moffitt’s stroma subtypes, we found that patients within Immune Class and the normal stroma subtype had the best survival rate, whereas patients within the Nonimmune Class and the other stroma subtypes had the worst survival rate (P = 0.012, **Supplementary Figure 4C**). Finally, we combined the immune molecular subtypes with tumor and stroma classifications for prognostic stratification. Patients within Immune Class and the classical stroma and basal tumor subtypes had the best overall survival rate (P = 0.024, **Supplementary Figure 4D**). Together, these results showed that the combination of immune molecular subtypes with tumor and/(or) stromal subtypes achieved a cumulative effect on PDAC prognosis prediction.

### Validation in Independent Datasets

**Figure 8:**
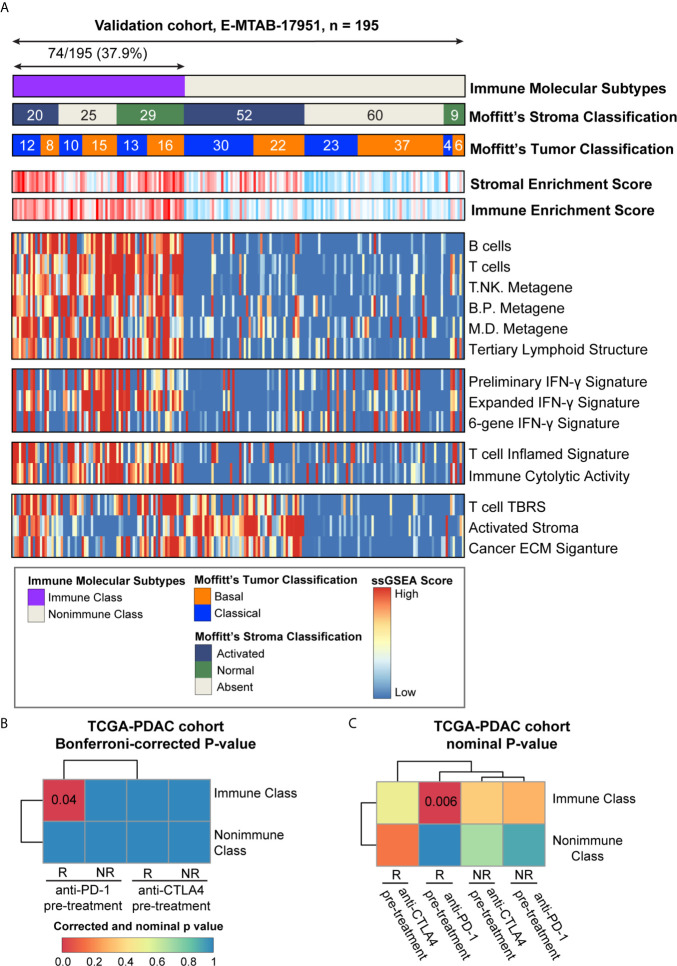
Analyses of potential immunotherapy response and validation in E-MTAB-17951. **(A)** The presence and molecular characteristics of the immune molecular subtypes were validated in cohort E-MTAB-17951. The heatmap showed the single sample gene set enrichment analysis (ssGSEA) scores of immune- and stroma- related signatures. Moffitt’s tumor/stroma classifications were also shown at the top panel. **(B)** SubMap analysis was used to evaluate the immune molecular subtypes in the TCGA-PDAC cohort and four groups of melanoma patients (pre-treatment CTLA-4 inhibitor responders and non-responders, pre-treatment PD-1 inhibitor responders and non-responders). Similarity between these two cohorts were illustrated as Bonferroni-corrected P-values. **(C)** SubMap analysis was used to evaluate the immune molecular subtypes in the TCGA-PDAC cohort and four groups of melanoma patients (pre-treatment CTLA-4 inhibitor responders and non-responders, pre-treatment PD-1 inhibitor responders and non-responders). Similarity between these two cohorts were illustrated as nominal P-values. PD-1, programmed cell death-1; CTLA-4, cytotoxic T lymphocyte-associated antigen-4; B/P metagene, B cell/plasma cell metagene; M/D metagene, monocyte/dendritic cell metagene; IFN-γ, interferon-γ; TBRS, transforming growth factor-β response signature; ECM, extracellular matrix.

The presence of immune molecular subtypes was further evaluated in five independent datasets using NTP analyses with the 100 gene-expression-based Immune Classifier (n = 615, **Figure 1** and **Supplementary Table 1**). Gene expression profiling of the validation datasets was conducted with different microarray platforms (Illumina, Affymetrix, or Agilent Gene chip systems) and in different types of tissue material (flash frozen or formalin fixed paraffin embedded). The proportion of patients classified as Immune Class showed consistency among the validation datasets, with an average of 36.4% (range 30.0%-42.8%) (**Figure 8A** and **Supplementary Figures 6–9**). Patients in validation cohort GSE57495 were allocated to Immune Class at a higher frequency of 42.8%, potentially due to the different microarray platforms used (Custom Affymetrix 2.0 microarray). Overall, the immune molecular subtypes were successfully reproduced in the validation datasets regardless of the platform and type of tumor tissue used.

### Exploration of Potential Immunotherapy Response

The ability of the immune molecular subtypes to predict immunotherapy response was explored using subclass mapping analysis. We assessed the similarity of immune-related gene expression profiles between the TCGA-PDAC cohort and a cohort of 32 melanoma patients receiving sequential CTLA-4 and PD-1 inhibitors (**Figures 8B, C**) (37, 50). Our results showed similarities between patients within Immune Class and melanoma patients responding to PD-1 checkpoint inhibitors (Bonferroni corrected p-value = 0.04). The similarity of immune-related gene expression profiles between Immune Class and immunotherapy responders was also shown in the validation cohorts (**Supplementary Figures 6–9**). To further explore this similarity, we included a cohort of 65 patients with non-small cell lung cancer (NSCLC), head and neck squamous cell carcinoma (HNSCC) and melanoma who were treated with PD-1 inhibitors (51). Significant similarity between Immune Class and PD-1 inhibitor responder was observed in the TCGA-PDAC cohort and the E-MTAB-17951 validation cohort (**Supplementary Figures 5A, B, E, F**). Thus, we showed the discrepant responses of immunotherapy in two immune molecular subtypes, which needs to be strengthened in PDAC patients receiving immune checkpoint inhibitors.

## Discussion

Immunotherapy, especially immune checkpoint inhibitors, has emerged as a new era of cancer treatment. Nevertheless, immune checkpoint inhibitors could only benefit a minority of PDAC patients (12). The limited clinical benefit of immune checkpoint inhibitors achieved in PDAC patients necessitates the identification of suitable PDAC patients. Deep understanding of the tumor immune microenvironment was also necessary in identifying such patients. In the current study, the NMF method was applied to deconvolute the gene expression profiles and identify immune molecular subtypes. We then discovered a robust immune molecular subtype, Immune Class, which comprised 30.8% of the cohort. Detailed immunogenomic profiling was conducted, and a comprehensive description of the tumor-, stromal-, immune- compartments was provided. The presence of Immune Class reflected an active immune response and correlated with current immunotherapy biomarkers.

In this study, we provided an immune molecular classification that, similar to current PDAC molecular classifications, has prognostic value in PDAC. Immune Class was an independent favorable prognostic factor, as confirmed in both the training and validation cohorts. Furthermore, in-depth survival analyses confirmed that integration of the immune molecular subtypes with Moffitt’s tumor and stroma classifications had a cumulative effect on prognosis prediction. According to Moffitt et al., the tumor and stroma classifications were similarly based on virtual microdissection of the tumor epithelium and stromal components with the NMF method (15). These findings suggest the complex interplay among the tumor, stromal and immune compartments and support combination therapeutic strategies targeting the tumor microenvironment. Upon comparison to a melanoma cohort, we found that our Immune Class was associated with melanoma patients responding to PD-1 inhibitors, suggesting its potential immunotherapy efficacy. Successful reproduction in five independent datasets suggested the robustness of the immune molecular subtypes. Liu et al. also identified immune classification of PDAC, but used a method of consensus clustering rather than NMF (52). NMF can separate tumor, stromal and immune gene expression from transcriptomic data to deconvolute context-dependent signals. Moreover, compared to their study, we used twice the sample size and conducted a more comprehensive analysis including other current immunotherapy biomarkers, such as TMB and neoantigen count. Our findings indicate the prognostic value of our classification, but further validation in PDAC patients receiving immune checkpoint blockade therapies is required.

Given that the tumor microenvironment of PDAC is comprised of an admixture of abundant stromal cells and immune cells, it is critical to consider tumor purity when interpreting the genomic and transcriptional profiles. Because of the modest concordance among intraplatform tumor purity estimates (40, 53), we compared the gold-standard pathologist-reviewed tumor cellularity with bioinformatic estimates, including ABSOLUTE purity, DNA methylation-estimated purity, and copy number-estimated purity. Nevertheless, in our study, DNA methylation-estimated tumor purity and ABSOLUTE purity were well correlated. Generally, Immune Class had lower tumor purity than Nonimmune Class, probably due to higher immune cell infiltration in Immune Class. Regarding prognosis prediction, the immune molecular subtypes rather than tumor purity served as a prognostic factor.

Multiple biomarkers of response to immunotherapy have been developed and include four main categories (1): antigens eliciting T cell responses, such as TMB, CNAs, and neoantigen counts (2); mechanisms of immune evasion, such as CTLA-4 and PD-L1 expression and certain oncogenic pathways (3); markers of immune infiltration, such as CD8+ T cell infiltration; and (4) host factors (54). In our study, both TMB and CNAs were associated with the immune molecular subtypes. Although PDAC has a relatively a lower TMB than other solid tumors (5), there was still a tendency for a higher TMB in Immune Class. We also concluded that patients within Immune Class had relatively lower both broad- and focal-level CNAs. These findings highlight increased genomic stability in Immune Class and the role of aneuploidy in regulating immune response. Nevertheless, an association between neoantigen counts and immune molecular subtypes was not identified, which might be explained by the fact that neoantigen quality, rather than neoantigen quantity, is responsible for the CD8+ T cell-mediated immune response (55). In addition, the expression of immunomodulatory genes was compared between immune molecular subtypes to infer the potential immune evasion mechanisms. The expression of immune checkpoint molecules, such as PD-L1 and CTLA-4, was higher in Immune Class. Other immunostimulatory or immunosuppressive genes, including inducible T Cell costimulator (*ICOS*), 2,3-dioxygenase 1 (*IDO1*), and selectin P (*SELP*), was also differentially expressed (13, 56, 57).

The molecular characteristics of Immune Class also included elevated immune cytolytic activity, IFN-γ signaling upregulation, and increased immune cell infiltration. Immune cytolytic activity, defined as the geometric mean of *GZMA* and perforin 1 (*PRF1*) expression, is associated with resistance to and relapse following immunomodulatory therapies (35). A six-gene IFN-γ signature that can be used to predict the response to pembrolizumab in melanoma patients was also significantly enriched in Immune Class. In previous research, IFN signaling was considered an important inducer of the innate and adaptive responses and served as a new therapeutic approach in pancreatic cancer. The upregulation of IFN signaling promoted PD-L1 expression, facilitated recruitment of CD8+ T cells and induced immunogenic cell death (34, 58). In addition, we also observed significant enrichment of both adaptive and innate immune cell subpopulations using ssGSEA and CIBERSORT. Cytolytic T cells and NK cells, together with a T cell inflamed signature, indicating the upregulation of cellular immunity, were enriched in Immune Class. Similarly, B cells and plasma cells, together with a B cell/plasma cell metagene, implying the upregulation of humoral immunity, were also enriched. Interestingly, the majority of TILs in PDAC were macrophages, indicating the potential of targeting tumor-associated macrophages. Immune Class had significantly more infiltration of proinflammatory M1 macrophages, whereas Nonimmune Class had more infiltration of anti-inflammatory M2 macrophages. These findings indicate upregulation of innate immune response in Immune Class.

In the current study, the enrichment of stromal signatures and upregulation of the TGF-β and WNT/β-catenin pathways were detected in Immune Class. Immune Class also had fewer mutations in the WNT/β-catenin pathway. It is well established that intrinsic tumor activation of the TGF-β pathway plays a role in the suppression of CD8+ T cell recruitment and function as well as the proliferation and activation of CAFs (59, 60). The TGF-β pathway might also lead to chemotherapy and immunotherapy resistance. In addition, our findings demonstrated that patients within Immune Class had a significantly lower frequency of mutations in *SMAD4*. The loss of SMAD4 was previously reported to regulate the cell cycle and promote tumor proliferation and indicated poor survival in PDAC patients (61, 62). Crosstalk between the WNT/β-catenin pathway and TGF-β/SMAD4 pathway in the tumor immune microenvironment was thus implied. In conclusion, all these findings suggested that the immunotherapy response in PDAC was modulated by a combination of tumor-intrinsic mechanisms (e.g., TMB, CNAs, immunomodulatory gene expression, and certain oncogenic pathways) and tumor-extrinsic mechanisms (e.g., TILs).

In summary, our study revealed robust immune molecular subtypes in PDAC that achieved better performance in capturing immune components than previous classifications. Immune molecular subtypes correlated with currently used immunotherapy biomarkers, which confirmed the reliability of our classification. The cumulative effect of tumor, immune, and stroma classifications on prognosis prediction was confirmed. Nevertheless, our findings still require further validation in large cohorts of early-stage and metastatic PDAC patients. Additionally, further investigation should be performed in PDAC patients receiving immune checkpoint blockade therapies, to demonstrate its potential value in the immunotherapy response.

## Data Availability Statement

The datasets generated in this study can be found in The Cancer Genome Atlas (pancreatic adenocarcinoma cohort), the Gene Expression Omnibus (GEO) (https://www.ncbi.nlm.nih.gov/geo/) under the accession numbers GSE85916, GSE71729, GSE57495, GSE21501, and Array Express (www.ebi.ac.uk/arraryexpress) under the accession number E-MTAB-17951 (**Supplementary Table 1**). 

## Author Contributions

RL was involved in methodology, software, formal analysis, visualization, data curation, and writing - original draft. YH was involved in software and writing - review and editing. HZ was involved in validation and data curation. JW was involved in validation and methodology. XL was involved in data curation and resources. HL was involved in validation and investigation. HW was involved in funding acquisition, methodology, project administration, and writing - review and editing. ZL was involved in supervision, conceptualization, funding acquisition, and project administration RL and HW have accessed verified the underlying data. All authors contributed to the article and approved the submitted version.

## Funding

This work was supported by Intergovernmental International Science, Technology and Innovation Cooperation Key Project of the National Key R&D Programme (NKP) (Project No.2017YFE0110300) to ZL, National Natural Science Foundation of China (Project No.82072749) to ZL, and National Natural Science Foundation of China (Project No.82072747) to HW.

## Conflict of Interest

The authors declare that the research was conducted in the absence of any commercial or financial relationships that could be construed as a potential conflict of interest.

## Abbreviations

PDAC, pancreatic ductal adenocarcinoma; TCGA, The Cancer Genome Atlas; NMF, non-negative matrix factorization; TMB, tumor mutational burden; CNAs, copy number alterations; PD-1, programmed cell death-1; QM, quasimesenchymal; ADEX, aberrantly differentiated endocrine exocrine; CAFs, cancer-associated fibroblasts; FPKM, fragments per kilobase of exon model per million mapped fragments; GEO, Gene Expression Omnibus; ssGSEA, single-sample gene set enrichment analysis; GO, Gene Ontology; KEGG, Kyoto Encyclopedia of Genes and Genomes; FDR, false discovery rate; MDS plot, multidimensional scaling plot; NTP, nearest template prediction; ESTIMATE, Estimation of Stromal and Immune cells in MAlignant Tumor tissues using Expression data; DEGs, differentially expressed genes; GSEA, gene set enrichment analysis; SMGs, significantly mutated genes; TILs, tumor-infiltrating lymphocytes; CoxPH regression model, Cox proportional hazards regression model; CTLA-4, cytotoxic T lymphocyte-associated antigen-4; NK cells, natural killer cells; PD-L1, programmed death ligand 1; TGF-β, transforming growth factor; TNF-α, necrosis factor-α; MHC, major histocompatibility complex; HR, hazard ratio.

## References

[1] Mizrahi JD Surana R Valle JW Shroff RT. Pancreatic Cancer. Lancet (2020) 395:2008–20. 10.1016/s0140-6736(20)30974-0 32593337

[2] Zhang S Sun K Zheng R Zeng H Wang S Chen R. Cancer Incidence and Mortality in China, 2015. J Natl Cancer Center (2020) 1:2–11. 10.1016/j.jncc.2020.12.001 PMC11256613 39036787

[3] Conroy T Hammel P Hebbar M Ben Abdelghani M Wei AC Raoul JL. FOLFIRINOX or Gemcitabine as Adjuvant Therapy for Pancreatic Cancer. N Engl J Med (2018) 379:2395–406. 10.1056/NEJMoa1809775 30575490

[4] Nevala-Plagemann C Hidalgo M Garrido-Laguna I. From State-of-the-Art Treatments to Novel Therapies for Advanced-Stage Pancreatic Cancer. Nat Rev Clin Oncol (2020) 17:108–23. 10.1038/s41571-019-0281-6 31705130

[5] Rizvi NA Hellmann MD Snyder A Kvistborg P Makarov V Havel JJ. Cancer Immunology. Mutational Landscape Determines Sensitivity to PD-1 Blockade in non-Small Cell Lung Cancer. Science (2015) 348:124–8. 10.1126/science.aaa1348 PMC4993154 25765070

[6] Le DT Uram JN Wang H Bartlett BR Kemberling H Eyring AD. PD-1 Blockade in Tumors With Mismatch-Repair Deficiency. N Engl J Med (2015) 372:2509–20. 10.1056/NEJMoa1500596 PMC4481136 26028255

[7] Patel SP Kurzrock R. PD-L1 Expression as a Predictive Biomarker in Cancer Immunotherapy. Mol Cancer Ther (2015) 14:847–56. 10.1158/1535-7163.MCT-14-0983 25695955

[8] Collisson EA Sadanandam A Olson P Gibb WJ Truitt M Gu S. Subtypes of Pancreatic Ductal Adenocarcinoma and Their Differing Responses to Therapy. Nat Med (2011) 17:500–3. 10.1038/nm.2344 PMC3755490 21460848

[9] Bailey P Chang DK Nones K Johns AL Patch AM Gingras MC. Genomic Analyses Identify Molecular Subtypes of Pancreatic Cancer. Nature (2016) 531:47–52. 10.1038/nature16965 26909576

[10] Martens S Lefesvre P Nicolle R Biankin AV Puleo F Van Laethem JL. Different Shades of Pancreatic Ductal Adenocarcinoma, Different Paths Towards Precision Therapeutic Applications. Ann Oncol (2019) 30:1428–36. 10.1093/annonc/mdz181 31161208

[11] Birnbaum DJ Finetti P Birnbaum D Mamessier E Bertucci F. Validation and Comparison of the Molecular Classifications of Pancreatic Carcinomas. Mol Cancer (2017) 16:168. 10.1186/s12943-017-0739-z 29110659 PMC5674743

[12] Hosein AN Brekken RA Maitra A. Pancreatic Cancer Stroma: An Update on Therapeutic Targeting Strategies. Nat Rev Gastroenterol Hepatol (2020) 17:487–505. 10.1038/s41575-020-0300-1 32393771 PMC8284850

[13] Ho WJ Jaffee EM Zheng L. The Tumour Microenvironment in Pancreatic Cancer - Clinical Challenges and Opportunities. Nat Rev Clin Oncol (2020) 17:527–40. 10.1038/s41571-020-0363-5 PMC7442729 32398706

[14] Brunet JP Tamayo P Golub TR Mesirov JP. Metagenes and Molecular Pattern Discovery Using Matrix Factorization. Proc Natl Acad Sci USA (2004) 101:4164–9. 10.1073/pnas.0308531101 PMC384712 15016911

[15] Moffitt RA Marayati R Flate EL Volmar KE Loeza SG Hoadley KA. Virtual Microdissection Identifies Distinct Tumor- and Stroma-Specific Subtypes of Pancreatic Ductal Adenocarcinoma. Nat Genet (2015) 47:1168–78. 10.1038/ng.3398 PMC4912058 26343385

[16] Gibney GT Weiner LM Atkins MB. Predictive Biomarkers for Checkpoint Inhibitor-Based Immunotherapy. Lancet Oncol (2016) 17:e542–51. 10.1016/S1470-2045(16)30406-5 PMC5702534 27924752

[17] Blum A Wang P Zenklusen JC. SnapShot: TCGA-Analyzed Tumors. Cell (2018) 173:530. 10.1016/j.cell.2018.03.059 29625059

[18] Ritchie ME Phipson B Wu D Hu Y Law CW Shi W. Limma Powers Differential Expression Analyses for RNA-Sequencing and Microarray Studies. Nucleic Acids Res (2015) 43:e47. 10.1093/nar/gkv007 25605792 PMC4402510

[19] Gaujoux R Seoighe C. A Flexible R Package for Nonnegative Matrix Factorization. BMC Bioinf (2010) 11:367. 10.1186/1471-2105-11-367 PMC2912887 20598126

[20] Yoshihara K Shahmoradgoli M Martinez E Vegesna R Kim H Torres-Garcia W. Inferring Tumour Purity and Stromal and Immune Cell Admixture From Expression Data. Nat Commun (2013) 4:2612. 10.1038/ncomms3612 24113773 PMC3826632

[21] Yu G Wang LG Han Y He QY. Clusterprofiler: An R Package for Comparing Biological Themes Among Gene Clusters. OMICS (2012) 16:284–7. 10.1089/omi.2011.0118 PMC3339379 22455463

[22] Gene Ontology C. Gene Ontology Consortium: Going Forward. Nucleic Acids Res (2015) 43:D1049–56. 10.1093/nar/gku1179 PMC4383973 25428369

[23] Kanehisa M Sato Y Kawashima M Furumichi M Tanabe M. KEGG as a Reference Resource for Gene and Protein Annotation. Nucleic Acids Res (2016) 44:D457–62. 10.1093/nar/gkv1070 PMC4702792 26476454

[24] Chen YP Wang YQ Lv JW Li YQ Chua MLK Le QT. Identification and Validation of Novel Microenvironment-Based Immune Molecular Subgroups of Head and Neck Squamous Cell Carcinoma: Implications for Immunotherapy. Ann Oncol (2019) 30:68–75. 10.1093/annonc/mdy470 30407504

[25] Hanzelmann S Castelo R Guinney J. GSVA: Gene Set Variation Analysis for Microarray and RNA-Seq Data. BMC Bioinf (2013) 14:7. 10.1186/1471-2105-14-7 PMC3618321 23323831

[26] Hoshida Y. Nearest Template Prediction: A Single-Sample-Based Flexible Class Prediction With Confidence Assessment. PloS One (2010) 5:e15543. 10.1371/journal.pone.0015543 21124904 PMC2990751

[27] Reich M Liefeld T Gould J Lerner J Tamayo P Mesirov JP. GenePattern 2.0. Nat Genet (2006) 38:500–1. 10.1038/ng0506-500 16642009

[28] Subramanian A Tamayo P Mootha VK Mukherjee S Ebert BL Gillette MA. Gene Set Enrichment Analysis: A Knowledge-Based Approach for Interpreting Genome-Wide Expression Profiles. Proc Natl Acad Sci USA (2005) 102:15545–50. 10.1073/pnas.0506580102 PMC1239896 16199517

[29] Lawrence MS Stojanov P Polak P Kryukov GV Cibulskis K Sivachenko A. Mutational Heterogeneity in Cancer and the Search for New Cancer-Associated Genes. Nature (2013) 499:214–8. 10.1038/nature12213 PMC3919509 23770567

[30] Mayakonda A Lin DC Assenov Y Plass C Koeffler HP. Maftools: Efficient and Comprehensive Analysis of Somatic Variants in Cancer. Genome Res (2018) 28:1747–56. 10.1101/gr.239244.118 PMC6211645 30341162

[31] Cancer Genome Atlas Research Network Electronic address aadhe Cancer Genome Atlas Research N. Integrated Genomic Characterization of Pancreatic Ductal Adenocarcinoma. Cancer Cell (2017) 32:185–203.e13. 10.1016/j.ccell.2017.07.007 28810144 PMC5964983

[32] Newman AM Liu CL Green MR Gentles AJ Feng W Xu Y. Robust Enumeration of Cell Subsets From Tissue Expression Profiles. Nat Methods (2015) 12:453–7. 10.1038/nmeth.3337 PMC4739640 25822800

[33] Thorsson V Gibbs DL Brown SD Wolf D Bortone DS Ou Yang TH. The Immune Landscape of Cancer. Immunity (2019) 51:411–2. 10.1016/j.immuni.2019.08.004 31433971

[34] Ayers M Lunceford J Nebozhyn M Murphy E Loboda A Kaufman DR. IFN-Gamma-Related mRNA Profile Predicts Clinical Response to PD-1 Blockade. J Clin Invest (2017) 127:2930–40. 10.1172/JCI91190 PMC5531419 28650338

[35] Rooney MS Shukla SA Wu CJ Getz G Hacohen N. Molecular and Genetic Properties of Tumors Associated With Local Immune Cytolytic Activity. Cell (2015) 160:48–61. 10.1016/j.cell.2014.12.033 25594174 PMC4856474

[36] Balachandran VP Luksza M Zhao JN Makarov V Moral JA Remark R. Identification of Unique Neoantigen Qualities in Long-Term Survivors of Pancreatic Cancer. Nature (2017) 551:512–6. 10.1038/nature24462 PMC6145146 29132146

[37] Roh W Chen PL Reuben A Spencer CN Prieto PA Miller JP. Integrated Molecular Analysis of Tumor Biopsies on Sequential CTLA-4 and PD-1 Blockade Reveals Markers of Response and Resistance. Sci Transl Med (2017) 9:eaah3560. 10.1126/scitranslmed.aah3560 28251903 PMC5819607

[38] Balli D Rech AJ Stanger BZ Vonderheide RH. Immune Cytolytic Activity Stratifies Molecular Subsets of Human Pancreatic Cancer. Clin Cancer Res (2017) 23:3129–38. 10.1158/1078-0432.CCR-16-2128 PMC12164831 28007776

[39] Nissim L Wu MR Pery E Binder-Nissim A Suzuki HI Stupp D. Synthetic RNA-Based Immunomodulatory Gene Circuits for Cancer Immunotherapy. Cell (2017) 171:1138–50.e15. 10.1016/j.cell.2017.09.049 29056342 PMC5986174

[40] Haider S Tyekucheva S Prandi D Fox NS Ahn J Xu AW. Systematic Assessment of Tumor Purity and Its Clinical Implications. JCO Precis Oncol (2020) 4:995–1005. 10.1200/PO.20.00016 PMC7529507 33015524

[41] Rhee JK Jung YC Kim KR Yoo J Kim J Lee YJ. Impact of Tumor Purity on Immune Gene Expression and Clustering Analyses Across Multiple Cancer Types. Cancer Immunol Res (2018) 6:87–97. 10.1158/2326-6066.CIR-17-0201 29141981

[42] Pan H Lu L Cui J Yang Y Wang Z Fan X. Immunological Analyses Reveal an Immune Subtype of Uveal Melanoma With a Poor Prognosis. Aging (Albany N Y) (2020) 12:1446–64. 10.18632/aging.102693 PMC7053626 31954372

[43] Puleo F Nicolle R Blum Y Cros J Marisa L Demetter P. Stratification of Pancreatic Ductal Adenocarcinomas Based on Tumor and Microenvironment Features. Gastroenterology (2018) 155:1999–2013.e3. 10.1053/j.gastro.2018.08.033 30165049

[44] Carter SL Cibulskis K Helman E McKenna A Shen H Zack T. Absolute Quantification of Somatic DNA Alterations in Human Cancer. Nat Biotechnol (2012) 30:413–21. 10.1038/nbt.2203 PMC4383288 22544022

[45] Nicolle R Blum Y Duconseil P Vanbrugghe C Brandone N Poizat F. Establishment of a Pancreatic Adenocarcinoma Molecular Gradient (PAMG) That Predicts the Clinical Outcome of Pancreatic Cancer. EBioMedicine (2020) 57:102858. 10.1016/j.ebiom.2020.102858 32629389 PMC7334821

[46] Dijk F Veenstra VL Soer EC Dings MPG Zhao L Halfwerk JB. Unsupervised Class Discovery in Pancreatic Ductal Adenocarcinoma Reveals Cell-Intrinsic Mesenchymal Features and High Concordance Between Existing Classification Systems. Sci Rep (2020) 10:337. 10.1038/s41598-019-56826-9 31941932 PMC6962149

[47] Connor AA Denroche RE Jang GH Timms L Kalimuthu SN Selander I. Association of Distinct Mutational Signatures With Correlates of Increased Immune Activity in Pancreatic Ductal Adenocarcinoma. JAMA Oncol (2017) 3:774–83. 10.1001/jamaoncol.2016.3916 PMC5824324 27768182

[48] Golan T O'Kane GM Denroche RE Raitses-Gurevich M Grant RC Holter S. Genomic Features and Classification of Homologous Recombination Deficient Pancreatic Ductal Adenocarcinoma. Gastroenterology (2021) 160:2119–32.e9. 10.1053/j.gastro.2021.01.220 33524400

[49] Espinet E Gu Z Imbusch CD Giese NA Büscher M Safavi M. Aggressive PDACs Show Hypomethylation of Repetitive Elements and the Execution of an Intrinsic IFN Program Linked to a Ductal Cell of Origin. Cancer Discovery (2021) 11:638–59. 10.1158/2159-8290.Cd-20-1202 PMC9216338 33060108

[50] Chen PL Roh W Reuben A Cooper ZA Spencer CN Prieto PA. Analysis of Immune Signatures in Longitudinal Tumor Samples Yields Insight Into Biomarkers of Response and Mechanisms of Resistance to Immune Checkpoint Blockade. Cancer Discovery (2016) 6:827–37. 10.1158/2159-8290.CD-15-1545 PMC5082984 27301722

[51] Prat A Navarro A Pare L Reguart N Galvan P Pascual T. Immune-Related Gene Expression Profiling After PD-1 Blockade in Non-Small Cell Lung Carcinoma, Head and Neck Squamous Cell Carcinoma, and Melanoma. Cancer Res (2017) 77:3540–50. 10.1158/0008-5472.CAN-16-3556 28487385

[52] Liu J Liu Q Zhang X Cui M Li T Zhang Y. Immune Subtyping for Pancreatic Cancer With Implication in Clinical Outcomes and Improving Immunotherapy. Cancer Cell Int (2021) 21:137. 10.1186/s12935-021-01824-z 33637086 PMC7908647

[53] Aran D Sirota M Butte AJ. Systematic Pan-Cancer Analysis of Tumour Purity. Nat Commun (2015) 6:8971. 10.1038/ncomms9971 26634437 PMC4671203

[54] Litchfield K Reading JL Puttick C Thakkar K Abbosh C Bentham R. Meta-Analysis of Tumor- and T Cell-Intrinsic Mechanisms of Sensitization to Checkpoint Inhibition. Cell (2021) 184:596–614.e14. 10.1016/j.cell.2021.01.002 33508232 PMC7933824

[55] McGranahan N Furness AJ Rosenthal R Ramskov S Lyngaa R Saini SK. Clonal Neoantigens Elicit T Cell Immunoreactivity and Sensitivity to Immune Checkpoint Blockade. Science (2016) 351:1463–9. 10.1126/science.aaf1490 PMC4984254 26940869

[56] Huang J Chen P Liu K Liu J Zhou B Wu R. CDK1/2/5 Inhibition Overcomes IFNG-Mediated Adaptive Immune Resistance in Pancreatic Cancer. Gut (2020) 70:890–9. 10.1136/gutjnl-2019-320441 32816920

[57] Zhai L Ladomersky E Lenzen A Nguyen B Patel R Lauing KL. IDO1 in Cancer: A Gemini of Immune Checkpoints. Cell Mol Immunol (2018) 15:447–57. 10.1038/cmi.2017.143 PMC6068130 29375124

[58] Blaauboer A Sideras K van Eijck CHJ Hofland LJ. Type I Interferons in Pancreatic Cancer and Development of New Therapeutic Approaches. Crit Rev Oncol Hematol (2020) 159:103204. 10.1016/j.critrevonc.2020.103204 33387625

[59] Vennin C Murphy KJ Morton JP Cox TR Pajic M Timpson P. Reshaping the Tumor Stroma for Treatment of Pancreatic Cancer. Gastroenterology (2018) 154:820–38. 10.1053/j.gastro.2017.11.280 29287624

[60] Hezel AF Deshpande V Zimmerman SM Contino G Alagesan B O'Dell MR. TGF-Beta and Alphavbeta6 Integrin Act in a Common Pathway to Suppress Pancreatic Cancer Progression. Cancer Res (2012) 72:4840–5. 10.1158/0008-5472.CAN-12-0634 PMC3764481 22787119

[61] Gurumurthy S Bardeesy N. Uncapping NF-kappaB Activity in Pancreatic Cancer. EMBO J (2011) 30:1–2. 10.1038/emboj.2010.324 21206509 PMC3020124

[62] Tascilar M Skinner HG Rosty C Sohn T Wilentz RE Offerhaus GJ. The SMAD4 Protein and Prognosis of Pancreatic Ductal Adenocarcinoma. Clin Cancer Res (2001) 7:4115–21. 11751510

