# Supplementary material for: Identification and Validation of Immune Molecular Subtypes in Pancreatic Ductal Adenocarcinoma: Implications for Prognosis and Immunotherapy
Source: Front Immunol. 2021 Jul 15;12:690056. doi: 10.3389/fimmu.2021.690056 (PMC8320597; doi:10.3389/fimmu.2021.690056)
Supplement: Supplementary file 1 [file DataSheet_1.docx]

Supplementary Material

# Supplementary Data

No supplementary data available.

# Supplementary Figures and Tables

## Supplementary Figures

**Captions**

**Supplementary Figure 1: Identification of the immune NMF factor.** (A) Non-negative matrix factorization (NMF, k = 9 factors) was used to deconvolve the gene expression profiles in the training cohort. One factor was shown to correlate with immune enrichment score and subsequently referred as the immune NMF factor (indicated in purple). The expression of exemplar genes was illustrated in the heatmap. (B-C) Barplots showed the enrichment of GO (B) and KEGG pathways (C) in exemplar genes. GO terms were classified according three ontology: biological process (BP), cellular component (CC), and molecular function (MF).

**Supplementary Figure 2: Gene set enrichment analysis (GSEA) of the immune molecular subtypes.** GSEA between Immune Class and Nonimmune Class confirmed enrichment of immune cells (A), interferon signaling (B), interleukin signaling (D), other immune-related signaling (C), and immune response (E) (P < 0.05, FDR < 0.05).

**Supplementary Figure 3: Correlations of the immune molecular subtypes with immunomodulatory gene expression and immune cell infiltration.** (A) Heatmaps of immunomodulatory gene expression. Average gene expression levels in Immune Class and Nonimmune Class were log2 transformed and are displayed as colors ranging from red to blue as shown in the key. The functions of immunomodulatory genes were annotated as immune checkpoint stimulatory/inhibitory/not available (NA). (B) The relative proportions of 22 immune cell subpopulations in PDAC-TCGA cohort were estimated by CIBERSORT.

**Supplementary Figure 4: Integration of tumor-, stromal-, and immune- based PDAC classifications in prognostic stratification.** (A) Venn diagram compared the classifier genes among Moffitt’s tumor classification, Moffitt’s stroma classification, and the immune molecular subtypes. (B) Kaplan-Meier curves of overall survival were plotted according to Moffitt’s stromal classification. (C) Kaplan-Meier curves of overall survival were plotted according to the immune molecular subtypes and Moffitt’s stroma classification. (D) Kaplan-Meier curves of overall survival were plotted according to immune molecular subtypes and Moffitt’s tumor/stroma classifications. Log-rank test was used to compare different Kaplan-Meier curves.

**Supplementary Figure 5: Analyses of immunotherapy response in the TCGA cohort and the E-MTAB-17951 cohort.** (A) SubMap analysis was used to evaluate similarity between the immune molecular subtypes in the TCGA-PDAC cohort and PD-1 inhibitor responders. Similarity between these two cohorts were illustrated as Bonferroni-corrected P values and nominal P-values. (C) SubMap analysis was used to evaluate similarity between the immune molecular subtypes in the E-MTAB-17951 cohort and four groups of melanoma patients (pre-treatment CTLA-4 inhibitor responders and non-responders, pre-treatment PD-1 inhibitor responders and non-responders). Similarity between these two cohorts were illustrated as Bonferroni-corrected P values and nominal P-values. (C) SubMap analysis was used to evaluate similarity between the immune molecular subtypes in the E-MTAB-17951 cohort and PD-1 inhibitor responders. Similarity between these two cohorts were illustrated as Bonferroni-corrected and nominal P-values.

**Supplementary Figure 6: Validation of the immune molecular subtypes in GSE85916.** (A) The presence and molecular characteristics of the immune molecular subtypes were validated in cohort GSE85916. The heatmap showed the single sample gene set enrichment analysis (ssGSEA) scores of immune- and stroma- related signatures. Moffitt’s tumor/stroma classifications were also shown at the top panel. (B) Kaplan-Meier curves of overall survival were plotted according to the immune molecular subtypes (log-rank test). (C) SubMap analysis was used to evaluate similarity between the immune molecular subtypes and four groups of melanoma patients (pre-treatment CTLA-4 inhibitor responders and non-responders, pre-treatment PD-1 inhibitor responders and non-responders). Similarity between these two cohorts were illustrated as Bonferroni-corrected and nominal P-values.

**Supplementary Figure 7: Validation of the immune molecular subtypes in GSE71729.** (A) The presence and molecular characteristics of the immune molecular subtypes were validated in cohort GSE71729. The heatmap showed the single sample gene set enrichment analysis (ssGSEA) scores of immune- and stroma- related signatures. Moffitt’s tumor/stroma classifications were also shown at the top panel. (B) SubMap analysis was used to evaluate similarity between the immune molecular subtypes and four groups of melanoma patients (pre-treatment CTLA-4 inhibitor responders and non-responders, pre-treatment PD-1 inhibitor responders and non-responders). Similarity between these two cohorts were illustrated as Bonferroni-corrected and nominal P-values.

**Supplementary Figure 8: Validation of the immune molecular subtypes in GSE21501.** (A) The presence and molecular characteristics of the immune molecular subtypes were validated in cohort GSE21501. The heatmap showed the single sample gene set enrichment analysis (ssGSEA) scores of immune- and stroma- related signatures. Moffitt’s tumor/stroma classifications were also shown at the top panel. (B) SubMap analysis was used to evaluate similarity between the immune molecular subtypes and four groups of melanoma patients (pre-treatment CTLA-4 inhibitor responders and non-responders, pre-treatment PD-1 inhibitor responders and non-responders). Similarity between these two cohorts were illustrated as Bonferroni-corrected and nominal P-values.

**Supplementary Figure 9: Validation of the immune molecular subtypes in GSE57495.** (A) The presence and molecular characteristics of the immune molecular subtypes were validated in cohort GSE57495. The heatmap showed the single sample gene set enrichment analysis (ssGSEA) scores of immune- and stroma- related signatures. Moffitt’s tumor/stroma classifications were also shown at the top panel. (B) SubMap analysis was used to evaluate similarity between the immune molecular subtypes and four groups of melanoma patients (pre-treatment CTLA-4 inhibitor responders and non-responders, pre-treatment PD-1 inhibitor responders and non-responders). Similarity between these two cohorts were illustrated as Bonferroni-corrected and nominal P-values.

## Supplementary Tables

**Supplementary Table 1:** Public datasets in the training cohort and the validation cohort

**Supplementary Table 2:** Coefficient matrix and basis matrix of NMF analysis

**Supplementary Table 3:** Details of exemplar genes

**Supplementary Table 4:** Publicly-available gene expression signatures used for single-cell gene set enrichment analysis

**Supplementary Table 5:** Results of GO and KEGG enrichment

**Supplementary Table 6:** Lists of genes significantly over-expressed in the Immune Class and Nonimmune Class

**Supplementary Table 7:** Details of Immune Classifier genes

**Supplementary Table 8:** GSEA results. Pathways enriched in Immune Class and Nonimmune Class.

**Supplementary Table 9:** Distribution of clinicopathological characteristics by the immune molecular subtypes

**Supplementary Table 10:** Distribution of tumor cellularity or purity by the immune molecular subtypes

**Supplementary Table 11:** Comparison of the immune molecular subtype with other pancreatic molecular classifications

**Supplementary Table 12:** Univariable Cox proportional hazard regression analysis of the immune molecular subtypes and other pancreatic molecular classifications

**Supplementary Table 1: Public datasets in the training cohort and the validation cohort**

| **Reference** | **Source of data** | **Technological platform** | **Sample collection** | **Sample Type** | **Total number of samples** | **Number of samples included in the present study** |
| --- | --- | --- | --- | --- | --- | --- |
| Cancer Genome Atlas Research Network et al. (1) | TCGA-PAAD | Illumina HiSeq RNA sequencing | Bulk | Primary PDAC | 178 | 149 |
| Jandaghi P et al. (2) | E-MTAB-1791 | Illumina human WG6 BeadChip v3 | Bulk | Primary PDAC, flash frozen | 457 | 195 |
| Moffitt RA et al. (3) | GEO database, GSE71729 | Agilent-014850 Whole Human Genome Microarray 4x44K G4112F | Bulk | Primary PDAC, flash frozen | 375 | 145 |
| Stratford JK et al. (4) | GEO database, GSE21501 | Agilent-014850 Whole Human Genome Microarray 4x44K G4112F | Bulk | Primary PDAC, flash frozen | 132 | 132 |
| Puleo F et al. (5) | GEO database, GSE85916 | [HG-U219] Affymetrix Human Genome U219 Array | Bulk | Treatment naïve primary PDAC tissue, FFPE | 80 | 80 |
| Chen DT et al. (6) | GEO database, GSE57495 | Rosetta/Merck Human RSTA Custom Affymetrix 2.0 microarray | Macrodissection | Primary PDAC, flash frozen | 63 | 63 |

**Supplementary Table 4: Publicly-available gene expression signatures used for single-cell gene set enrichment analysis**

| **Gene expression signatures** | **Reference** |
| --- | --- |
| Immune enrichment score | Yoshihara K, et al. Nat Commun. 2013;4: 2612 (7) |
| Stromal enrichment score | Yoshihara K, et al. Nat Commun. 2013;4: 2612 (7) |
| B cells | Bindea G, et al. Immunity. 2013;39:782-95 (8) |
| T cells | Bindea G, et al. Immunity. 2013;39:782-95 (8) |
| T helper cells | Bindea G, et al. Immunity. 2013;39:782-95 (8) |
| Central memory T (Tcm) cells | Bindea G, et al. Immunity. 2013;39:782-95 (8) |
| Effector memory T (Tem) cells | Bindea G, et al. Immunity. 2013;39:782-95 (8) |
| Type 1 helper T (Th1) cells | Bindea G, et al. Immunity. 2013;39:782-95 (8) |
| Type 2 helper T (Th2) cells | Bindea G, et al. Immunity. 2013;39:782-95 (8) |
| T follicular helper (TFH) cells | Bindea G, et al. Immunity. 2013;39:782-95 (8) |
| T helper 17 (Th17) cells | Bindea G, et al. Immunity. 2013;39:782-95 (8) |
| Regulatory T (Treg) cells | Bindea G, et al. Immunity. 2013;39:782-95 (8) |
| CD8^+^ T cells | Bindea G, et al. Immunity. 2013;39:782-95 (8) |
| γδ T (Tgd) cells | Bindea G, et al. Immunity. 2013;39:782-95 (8) |
| Cytotoxic cells | Bindea G, et al. Immunity. 2013;39:782-95 (8) |
| Natural killer (NK) cells | Bindea G, et al. Immunity. 2013;39:782-95 (8) |
| NK CD57^dim^ cells | Bindea G, et al. Immunity. 2013;39:782-95 (8) |
| NK CD57^bright^ cells | Bindea G, et al. Immunity. 2013;39:782-95 (8) |
| Dendritic cells (DC) | Bindea G, et al. Immunity. 2013;39:782-95 (8) |
| Immature DC | Bindea G, et al. Immunity. 2013;39:782-95 (8) |
| Activated DC | Bindea G, et al. Immunity. 2013;39:782-95 (8) |
| Plasmacytoid DC | Bindea G, et al. Immunity. 2013;39:782-95 (8) |
| Eosinophils | Bindea G, et al. Immunity. 2013;39:782-95 (8) |
| Macrophages | Bindea G, et al. Immunity. 2013;39:782-95 (8) |
| Mast cells | Bindea G, et al. Immunity. 2013;39:782-95 (8) |
| Neutrophils | Bindea G, et al. Immunity. 2013;39:782-95 (8) |
| Major histocompatibility complex class II (MHC II) | Forero A, et al. Cancer Immunology Research. 2016;4(5):390-9 (9) |
| T cell/NK cell (T/NK) metagene | Alistar A, et al. Genome Med. 2014;6(10):80 (10) |
| B cell/Plasma cell (B/P) metagene | Alistar A, et al. Genome Med. 2014;6(10):80 (10) |
| Monocyte/Dendritic cell (M/D) metagene | Alistar A, et al. Genome Med. 2014;6(10):80 (10) |
| TITR UP/DOWN signature | Magnuson AM, et al. Proc Natl Acad Sci U S A. 2018;115(45):E10672-E81 (11) |
| M1 macrophage signature | Coates PJ, et al. Cancer Res. 2008;68(2):450-6 (12) |
| M2 macrophage signature | Coates PJ, et al. Cancer Res. 2008;68(2):450-6 (12) |
| Tertiary lymphoid structure (TLS) | Finkin S, et al. Nat Immunol. 2015;16(12):1235-44 (13) |
| T cell inflamed signature | Ribas A, et al. J Clin Oncol. 2015;33(15_suppl):3001 (14) |
| Immune cytolytic activity | Rooney MS, et al. Cell. 2015;160(1-2):48-61 (15) |
| Preliminary IFN-γ signature | Ayers M, et al. J Clin Invest. 2017;127(8):2930-40 (16) |
| Expanded IFN-γ signature | Ayers M, et al. J Clin Invest. 2017;127(8):2930-40 (16) |
| 6-gene IFN-γ signature | Ayers M, et al. J Clin Invest. 2017;127(8):2930-40 (16) |
| WNT/TGF-β UP/DOWN signature | Lachenmayer A, et al. Clin Cancer Res. 2012;18(18):4997-5007 (17) |
| T cell TGF-β response signature (TBRS) | Calon A, et al. Cancer Cell. 2012;22(5):571-84 (18) |
| Fibroblast TGF-β response signature (TBRS) | Calon A, et al. Cancer Cell. 2012;22(5):571-84 (18) |
| Macrophage TGF-β response signature (TBRS) | Calon A, et al. Cancer Cell. 2012;22(5):571-84 (18) |
| Endothelial TGF-β response signature (TBRS) | Calon A, et al. Cancer Cell. 2012;22(5):571-84 (18) |
| Cancer extracellular matrix (ECM) signature | Chakravarthy A, et al. Nat Commun. 2018;9(1):4692 (19) |

**Supplementary Table 9: Distribution of clinicopathological characteristics by immune molecular subtypes**

| **Variables** | **ALL** | **Immune Class** | **Nonimmune Class** | **P value** |
| --- | --- | --- | --- | --- |
|  | ***N=149 (Median / percent of whole cohort)*** | ***N=46 (Median / percent of Immune Class)*** | ***N=103 (Median / percent of Nonimmune Class)*** |  |
| **Age** | 65.0 (57.0;73.0) | 65.5 (56.2;73.8) | 65.0 (58.5;73.0) | 0.747 |
| **Sex** |  |  |  | 0.135 |
| Female | 69 (46.3%) | 26 (56.5%) | 43 (41.7%) |  |
| Male | 80 (53.7%) | 20 (43.5%) | 60 (58.3%) |  |
| **pTNM Stage** |  |  |  | 0.699 |
| Stage I/II | 140 (94.6%) | 42 (93.3%) | 98 (95.1%) |  |
| Stage III/IV | 8 (5.41%) | 3 (6.67%) | 5 (4.85%) |  |
| **Primary Tumor (T Stage)** |  |  |  | **0.035** |
| T1/T2 | 21 (14.2%) | 7 (15.6%) | 14 (13.6%) |  |
| T3 | 124 (83.8%) | 35 (77.8%) | 89 (86.4%) |  |
| T4 | 3 (2.03%) | 3 (6.67%) | 0 (0.00%) |  |
| **Lymph Nodes (N Stage)** |  |  |  | 0.890 |
| Negative | 39 (26.2%) | 13 (28.3%) | 26 (25.2%) |  |
| Positive | 109 (73.2%) | 33 (71.7%) | 76 (73.8%) |  |
| **Distant Metastasis (M Stage)** |  |  |  | 0.558 |
| Negative | 67 (45.0%) | 21 (45.7%) | 46 (44.7%) |  |
| Positive | 4 (2.68%) | 0 (0.00%) | 4 (3.88%) |  |
| Not measurable | 78 (52.3%) | 25 (54.3%) | 53 (51.5%) |  |
| **Histological Grade** |  |  |  | 0.294 |
| Moderate | 75 (50.3%) | 25 (54.3%) | 50 (48.5%) |  |
| Poor | 68 (45.6%) | 21 (45.7%) | 47 (45.6%) |  |
| Others | 6 (4.03%) | 0 (0.00%) | 6 (5.83%) |  |
| **Primary Site** |  |  |  | **0.049** |
| Head | 117 (78.5%) | 39 (84.8%) | 78 (75.7%) |  |
| Tail or body | 24 (16.1%) | 3 (6.52%) | 21 (20.4%) |  |
| Others | 8 (5.37%) | 4 (8.70%) | 4 (3.88%) |  |
| **Surgical Procedure** |  |  |  | 0.086 |
| Whipple | 116 (86.6%) | 40 (95.2%) | 76 (82.6%) |  |
| Distal pancreatectomy | 18 (13.4%) | 2 (4.76%) | 16 (17.4%) |  |
| **History of Chronic Pancreatitis** | |  |  | 0.511 |
| No | 107 (89.2%) | 31 (93.9%) | 76 (87.4%) |  |
| Yes | 13 (10.8%) | 2 (6.06%) | 11 (12.6%) |  |

**Supplementary Table 10: Distribution of tumor cellularity or purity by immune molecular subtypes**

|  | **ALL** | **Immune Class** | **Nonimmune Class** | **P value** |
| --- | --- | --- | --- | --- |
| **Variables** | ***N=149*** | ***N=46*** | ***N=103*** |  |
| **Pathological Tumor Cellularity** | 0.18 (10.0;25.0) | 0.12 (7.71;20.8) | 0.20 (11.8;27.9) | **0.005** |
| **ABSOLUTE Purity** | 0.34 (0.20;0.51) | 0.18 (0.13;0.25) | 0.38 (0.27;0.55) | **<0.001** |
| **DNA Hypermethylation Purity** | 0.40 (0.29;0.49) | 0.28 (0.22;0.37) | 0.45 (0.37;0.52) | **<0.001** |
| **Purity Class** |  |  |  | **<0.001** |
| Low | 73 (49.0%) | 37 (80.4%) | 36 (35.0%) |  |
| High | 76 (51.0%) | 9 (19.6%) | 67 (65.0%) |  |

**Supplementary Table 11: Comparison of the immune molecular subtype with other pancreatic molecular classifications**

|  | **ALL** | **Immune Class** | **Nonimmune Class** | **P value** |
| --- | --- | --- | --- | --- |
| **Variables** | ***N=149*** | ***N=46*** | ***N=103*** |  |
| **Moffitt’s Tumor Classification** |  |  |  | 0.384 |
| Basal | 65 (43.6%) | 23 (50.0%) | 42 (40.8%) |  |
| Classical | 84 (56.4%) | 23 (50.0%) | 61 (59.2%) |  |
| **Collison’s Classification** |  |  |  | **0.004** |
| Classical | 53 (35.6%) | 8 (17.4%) | 45 (43.7%) |  |
| Exocrine | 62 (41.6%) | 22 (47.8%) | 40 (38.8%) |  |
| QM | 34 (22.8%) | 16 (34.8%) | 18 (17.5%) |  |
| **Bailey’s Classification** |  |  |  | **<0.001** |
| Squamous | 31 (20.8%) | 5 (10.9%) | 26 (25.2%) |  |
| Immunogenic | 27 (18.1%) | 21 (45.7%) | 6 (5.83%) |  |
| Progenitor | 53 (35.6%) | 5 (10.9%) | 48 (46.6%) |  |
| ADEX | 38 (25.5%) | 15 (32.6%) | 23 (22.3%) |  |
| **Moffitt’s Stromal Classification** |  |  |  | **<0.001** |
| Activated | 49 (32.9%) | 11 (23.9%) | 38 (36.9%) |  |
| Absent | 71 (47.7%) | 15 (32.6%) | 56 (54.4%) |  |
| Normal | 29 (19.5%) | 20 (43.4%) | 9 (8.74%) |  |

**Supplementary Table 12: Univariable Cox proportional hazard regression analysis of the immune molecular subtypes and other pancreatic molecular classifications**

| **Variables** | **Univariable** | |
| --- | --- | --- |
|  | **HR (95% CI for HR)** | **P value** |
| **Immune Molecular Subtypes** |  |  |
| Nonimmune Class | 1.00 | - |
| Immune Class | 0.56 (0.33-0.95) | **0.033** |
| **Moffitt’s Tumor Classification** |  |  |
| Basal | 1.00 | - |
| Classical | 0.68 (0.43-1.09) | 0.107 |
| **Collison’s Classification** |  |  |
| Classical | 1.00 | - |
| Exocrine | 0.86 (0.51-1.44) | 0.560 |
| QM | 1.19 (0.67-2.12) | 0.551 |
| **Bailey’s Classification** |  |  |
| Squamous | 1.00 | - |
| Immunogenic | 0.58 (0.26-1.27) | 0.169 |
| Progenitor | 0.97 (0.55-1.70) | 0.912 |
| ADEX | 0.78 (0.41-1.49) | 0.449 |
| **Moffitt’s Stroma Classification** |  |  |
| Absent | 1.00 | - |
| Activated | 0.85 (0.52-1.41) | 0.535 |
| Normal | 0.46 (0.24-0.93) | **0.030** |

**References**

1. Cancer Genome Atlas Research Network. Electronic address aadhe, Cancer Genome Atlas Research N. Integrated Genomic Characterization of Pancreatic Ductal Adenocarcinoma. *Cancer Cell*. (2017) 32:185-203 e13. doi: 10.1016/j.ccell.2017.07.007

2. Jandaghi P, Najafabadi HS, Bauer AS, Papadakis AI, Fassan M, Hall A, et al. Expression of DRD2 Is Increased in Human Pancreatic Ductal Adenocarcinoma and Inhibitors Slow Tumor Growth in Mice. *Gastroenterology*. (2016) 151:1218-31. doi: 10.1053/j.gastro.2016.08.040

3. Moffitt RA, Marayati R, Flate EL, Volmar KE, Loeza SG, Hoadley KA, et al. Virtual microdissection identifies distinct tumor- and stroma-specific subtypes of pancreatic ductal adenocarcinoma. *Nat Genet*. (2015) 47:1168-78. doi: 10.1038/ng.3398

4. Stratford JK, Bentrem DJ, Anderson JM, Fan C, Volmar KA, Marron JS, et al. A six-gene signature predicts survival of patients with localized pancreatic ductal adenocarcinoma. *PLoS Med*. (2010) 7:e1000307. doi: 10.1371/journal.pmed.1000307

5. [Available from: <https://www.ncbi.nlm.nih.gov/geo/query/acc.cgi?acc=GSE85916>.

6. Chen DT, Davis-Yadley AH, Huang PY, Husain K, Centeno BA, Permuth-Wey J, et al. Prognostic Fifteen-Gene Signature for Early Stage Pancreatic Ductal Adenocarcinoma. *PLoS One*. (2015) 10:e0133562. doi: 10.1371/journal.pone.0133562

7. Yoshihara K, Shahmoradgoli M, Martinez E, Vegesna R, Kim H, Torres-Garcia W, et al. Inferring tumour purity and stromal and immune cell admixture from expression data. *Nat Commun*. (2013) 4:2612. doi: 10.1038/ncomms3612

8. Bindea G, Mlecnik B, Tosolini M, Kirilovsky A, Waldner M, Obenauf AC, et al. Spatiotemporal dynamics of intratumoral immune cells reveal the immune landscape in human cancer. *Immunity*. (2013) 39:782-95. doi: 10.1016/j.immuni.2013.10.003

9. Forero A, Li Y, Chen D, Grizzle WE, Updike KL, Merz ND, et al. Expression of the MHC Class II Pathway in Triple-Negative Breast Cancer Tumor Cells Is Associated with a Good Prognosis and Infiltrating Lymphocytes. *Cancer Immunology Research*. (2016) 4:390-9. doi: 10.1158/2326-6066.Cir-15-0243

10. Alistar A, Chou JW, Nagalla S, Black MA, D'Agostino R, Jr., Miller LD. Dual roles for immune metagenes in breast cancer prognosis and therapy prediction. *Genome Med*. (2014) 6:80. doi: 10.1186/s13073-014-0080-8

11. Magnuson AM, Kiner E, Ergun A, Park JS, Asinovski N, Ortiz-Lopez A, et al. Identification and validation of a tumor-infiltrating Treg transcriptional signature conserved across species and tumor types. *Proc Natl Acad Sci U S A*. (2018) 115:E10672-E81. doi: 10.1073/pnas.1810580115

12. Coates PJ, Rundle JK, Lorimore SA, Wright EG. Indirect macrophage responses to ionizing radiation: implications for genotype-dependent bystander signaling. *Cancer Res*. (2008) 68:450-6. doi: 10.1158/0008-5472.CAN-07-3050

13. Finkin S, Yuan D, Stein I, Taniguchi K, Weber A, Unger K, et al. Ectopic lymphoid structures function as microniches for tumor progenitor cells in hepatocellular carcinoma. *Nat Immunol*. (2015) 16:1235-44. doi: 10.1038/ni.3290

14. Ribas A, Robert C, Hodi FS, Wolchok JD, Joshua AM, Hwu W-J, et al. Association of response to programmed death receptor 1 (PD-1) blockade with pembrolizumab (MK-3475) with an interferon-inflammatory immune gene signature. *J Clin Oncol*. (2015) 33:3001-. doi: 10.1200/jco.2015.33.15_suppl.3001

15. Rooney MS, Shukla SA, Wu CJ, Getz G, Hacohen N. Molecular and genetic properties of tumors associated with local immune cytolytic activity. *Cell*. (2015) 160:48-61. doi: 10.1016/j.cell.2014.12.033

16. Ayers M, Lunceford J, Nebozhyn M, Murphy E, Loboda A, Kaufman DR, et al. IFN-gamma-related mRNA profile predicts clinical response to PD-1 blockade. *J Clin Invest*. (2017) 127:2930-40. doi: 10.1172/JCI91190

17. Lachenmayer A, Alsinet C, Savic R, Cabellos L, Toffanin S, Hoshida Y, et al. Wnt-pathway activation in two molecular classes of hepatocellular carcinoma and experimental modulation by sorafenib. *Clin Cancer Res*. (2012) 18:4997-5007. doi: 10.1158/1078-0432.CCR-11-2322

18. Calon A, Espinet E, Palomo-Ponce S, Tauriello DV, Iglesias M, Cespedes MV, et al. Dependency of colorectal cancer on a TGF-beta-driven program in stromal cells for metastasis initiation. *Cancer Cell*. (2012) 22:571-84. doi: 10.1016/j.ccr.2012.08.013

19. Chakravarthy A, Khan L, Bensler NP, Bose P, De Carvalho DD. TGF-beta-associated extracellular matrix genes link cancer-associated fibroblasts to immune evasion and immunotherapy failure. *Nat Commun*. (2018) 9:4692. doi: 10.1038/s41467-018-06654-8
