# Supplementary figures and images for: Identification and Validation of Immune Molecular Subtypes in Pancreatic Ductal Adenocarcinoma: Implications for Prognosis and Immunotherapy
Source: Front Immunol. 2021 Jul 15;12:690056. doi: 10.3389/fimmu.2021.690056 (PMC8320597; doi:10.3389/fimmu.2021.690056)

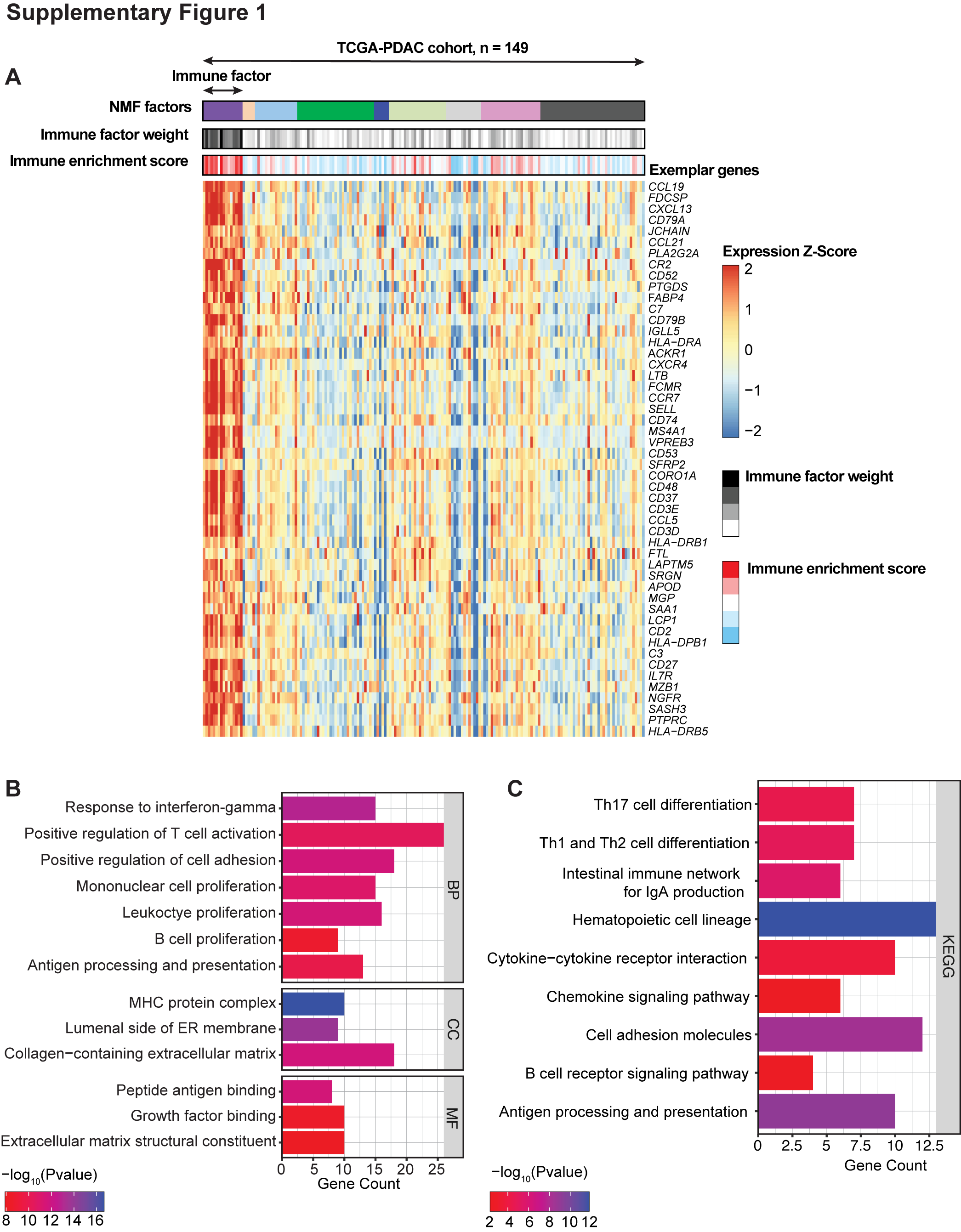

Supplement: Supplementary file 2 [file Image_1.tif]

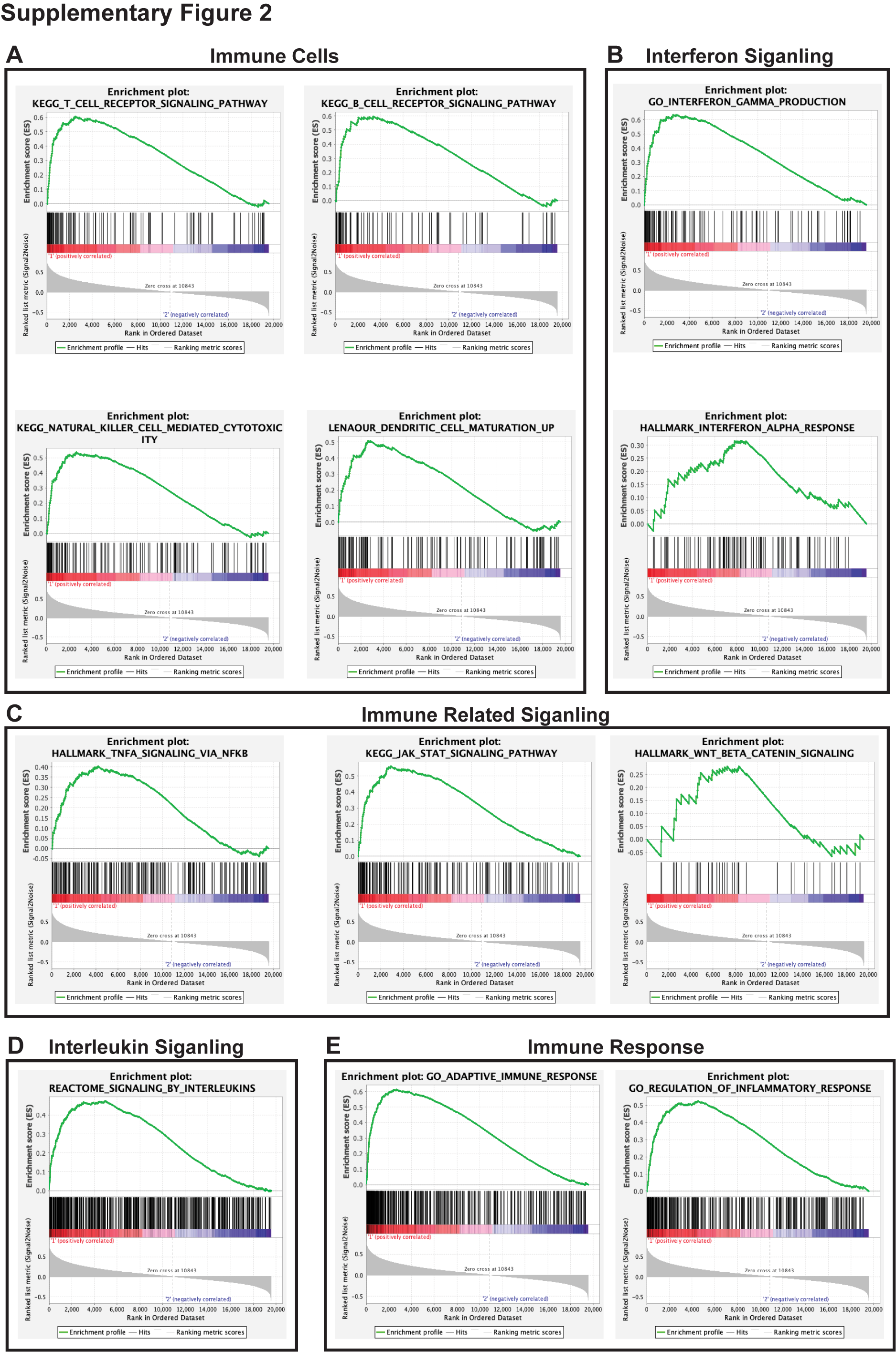

Supplement: Supplementary file 3 [file Image_2.tif]

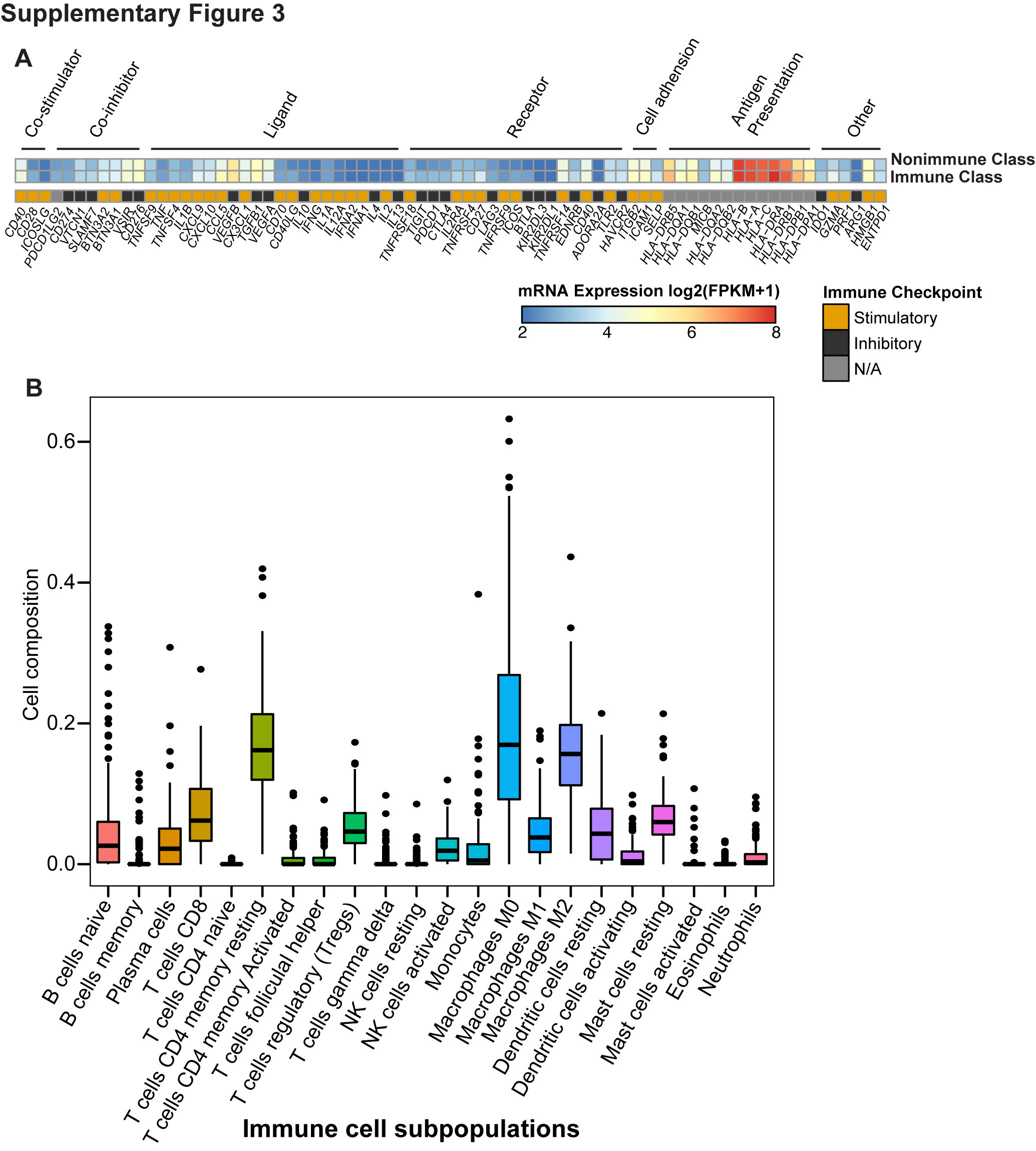

Supplement: Supplementary file 4 [file Image_3.tif]

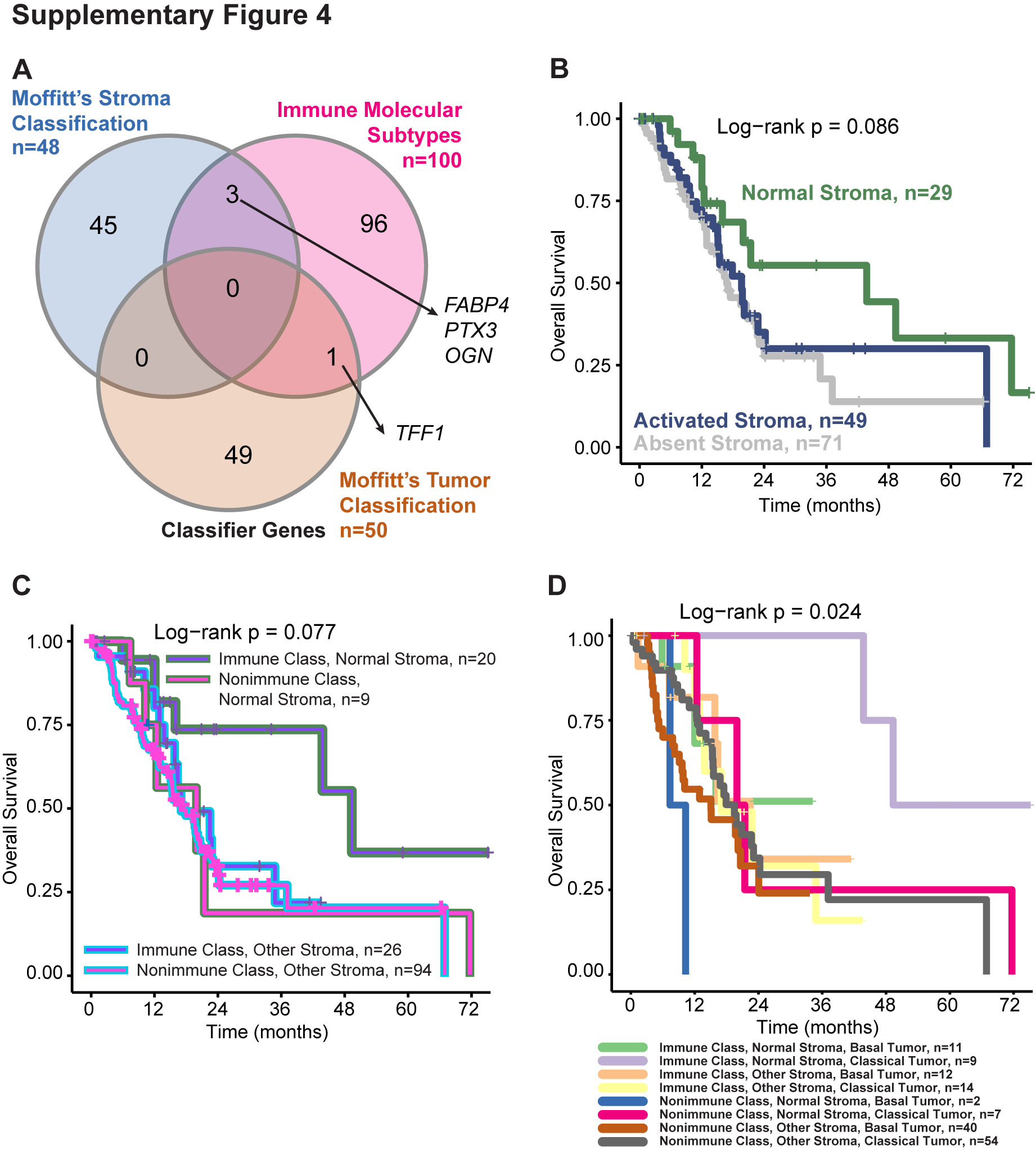

Supplement: Supplementary file 5 [file Image_4.tif]

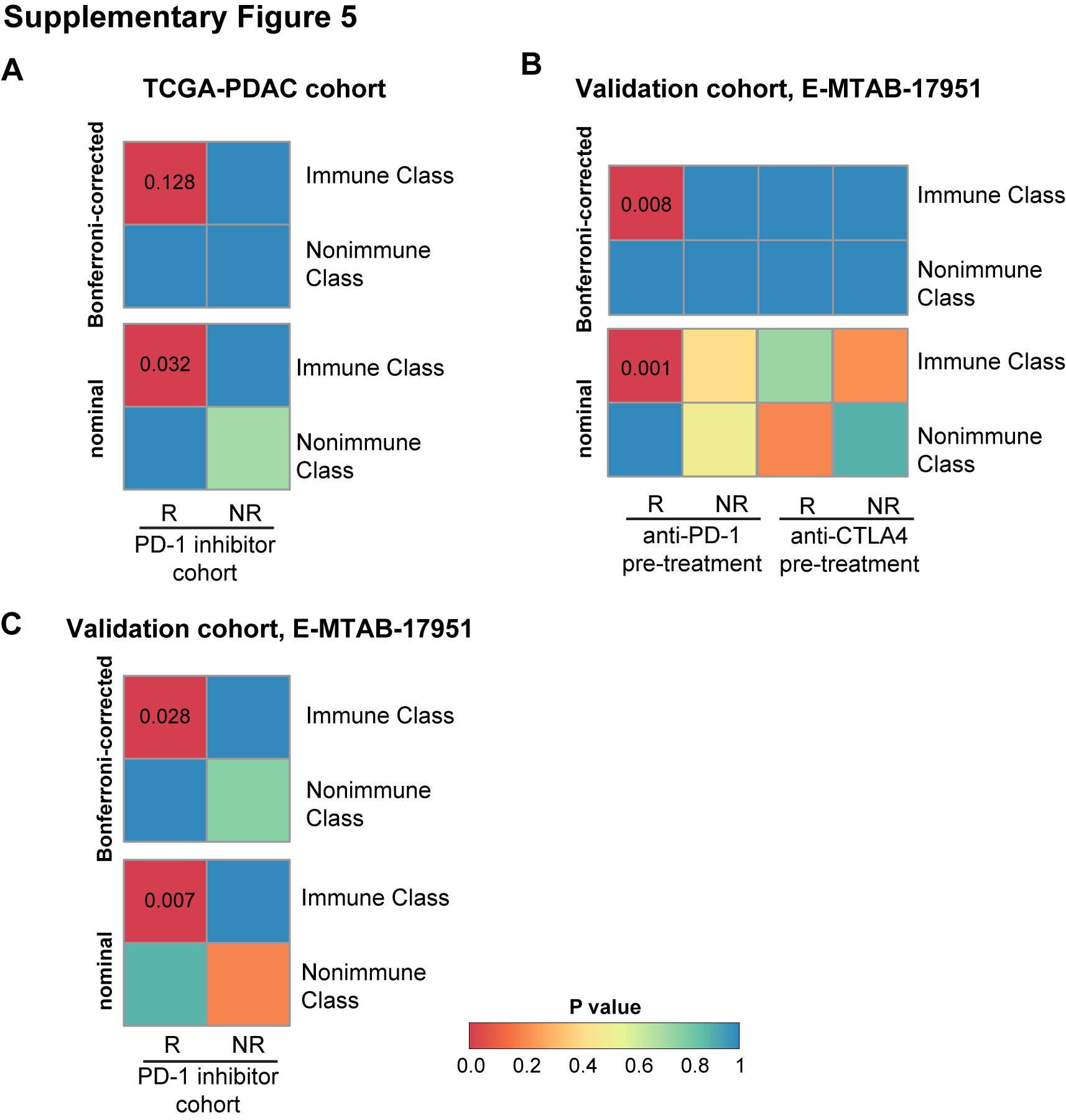

Supplement: Supplementary file 6 [file Image_5.tif]

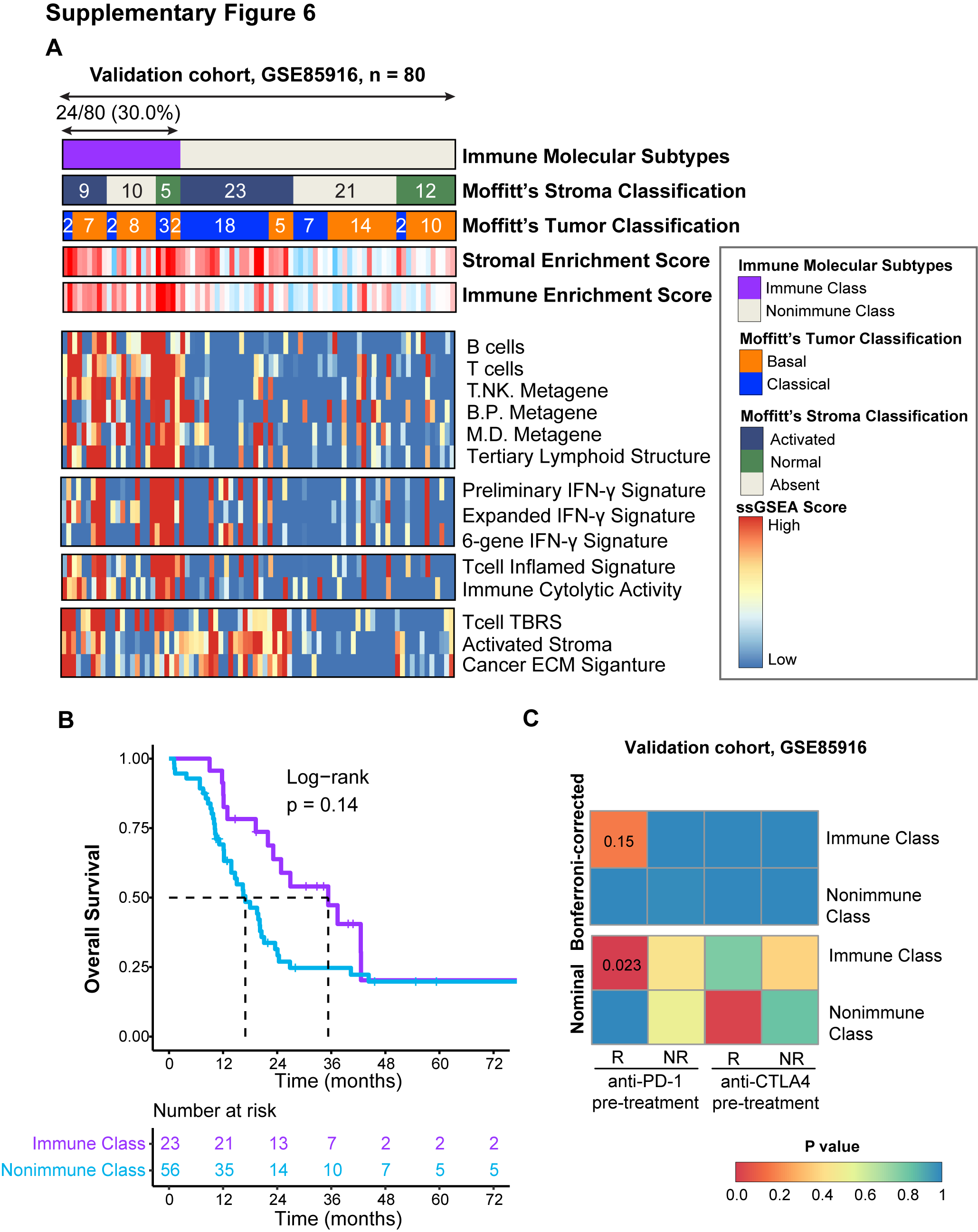

Supplement: Supplementary file 7 [file Image_6.tif]

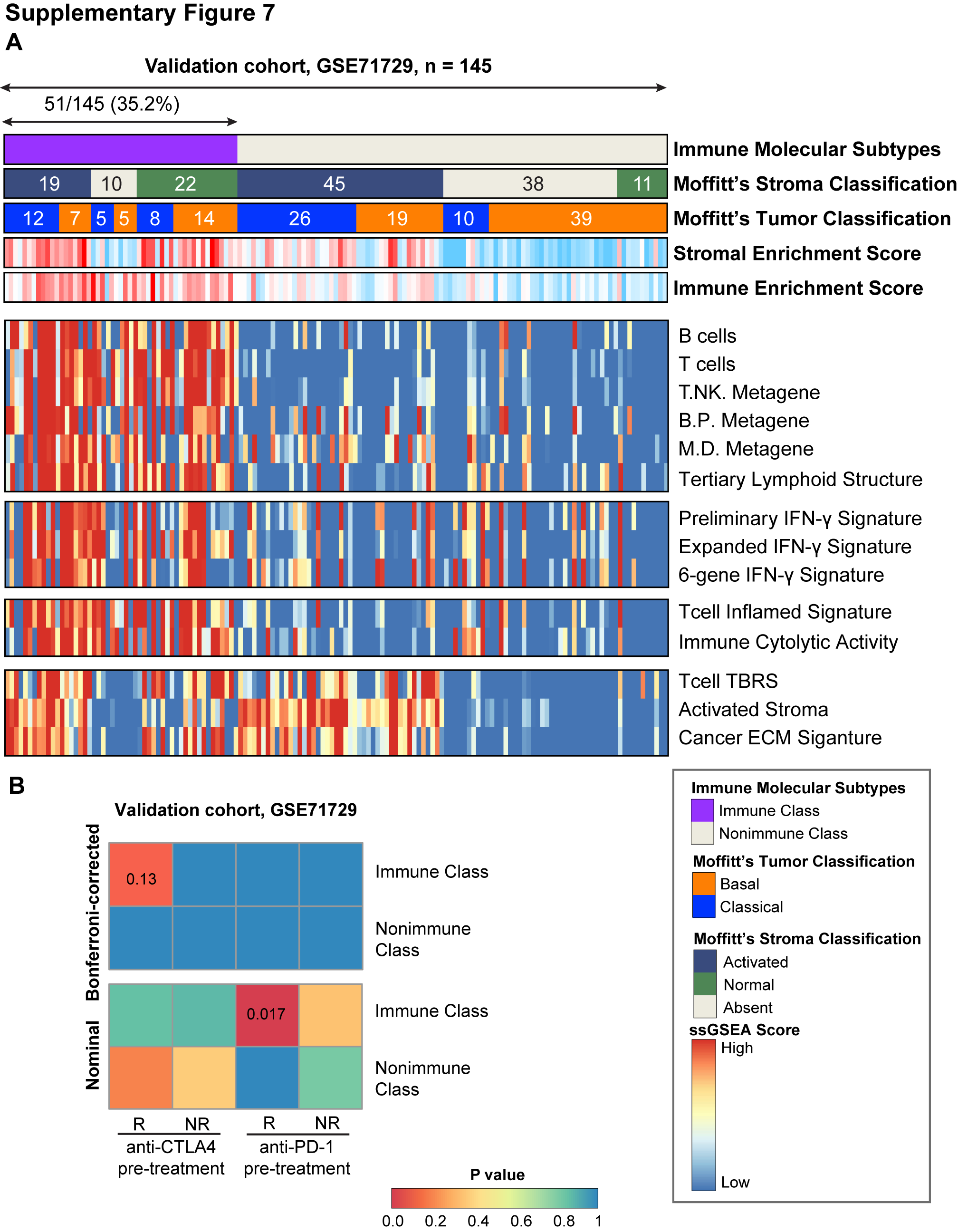

Supplement: Supplementary file 8 [file Image_7.tif]

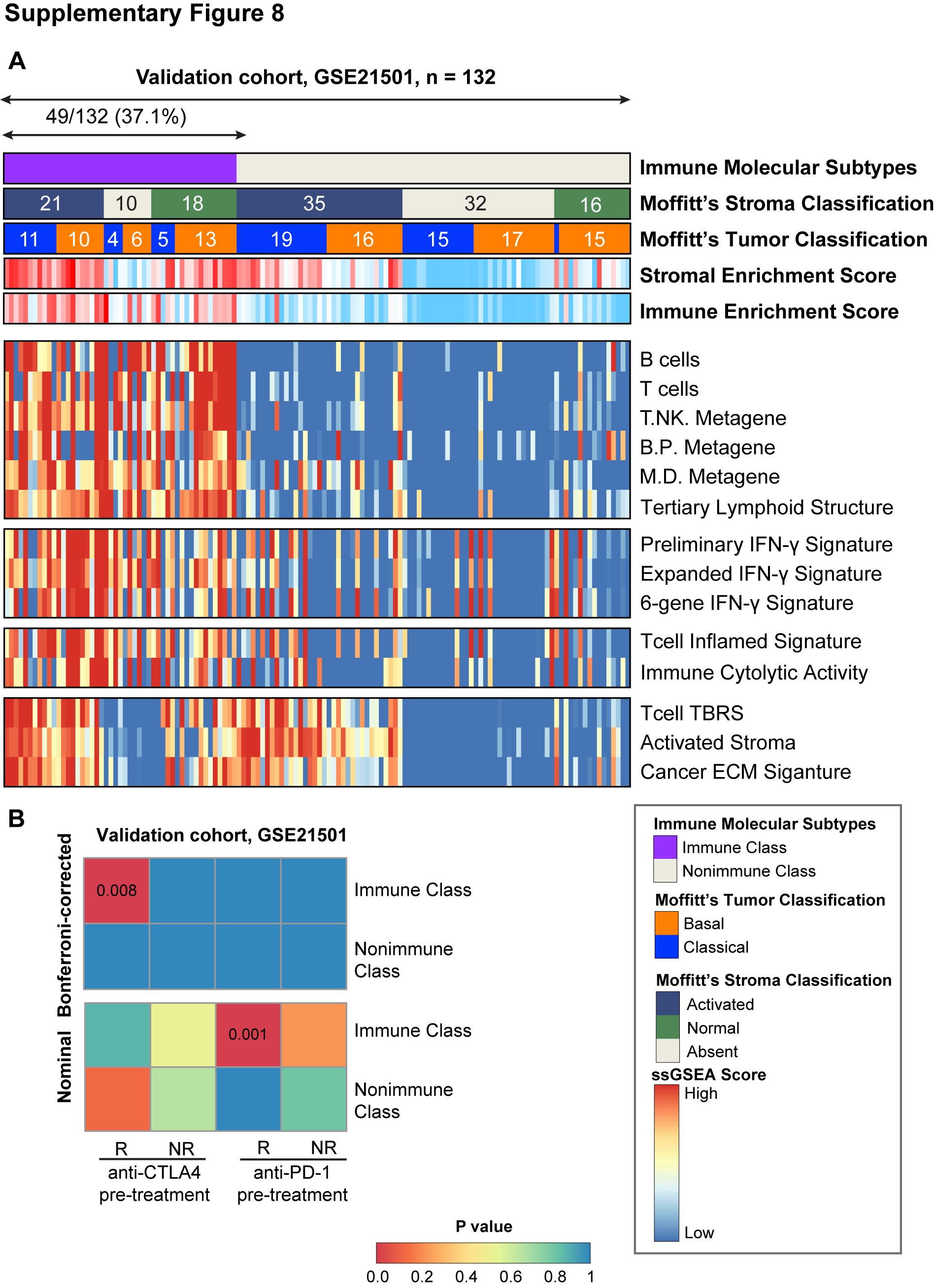

Supplement: Supplementary file 9 [file Image_8.tif]

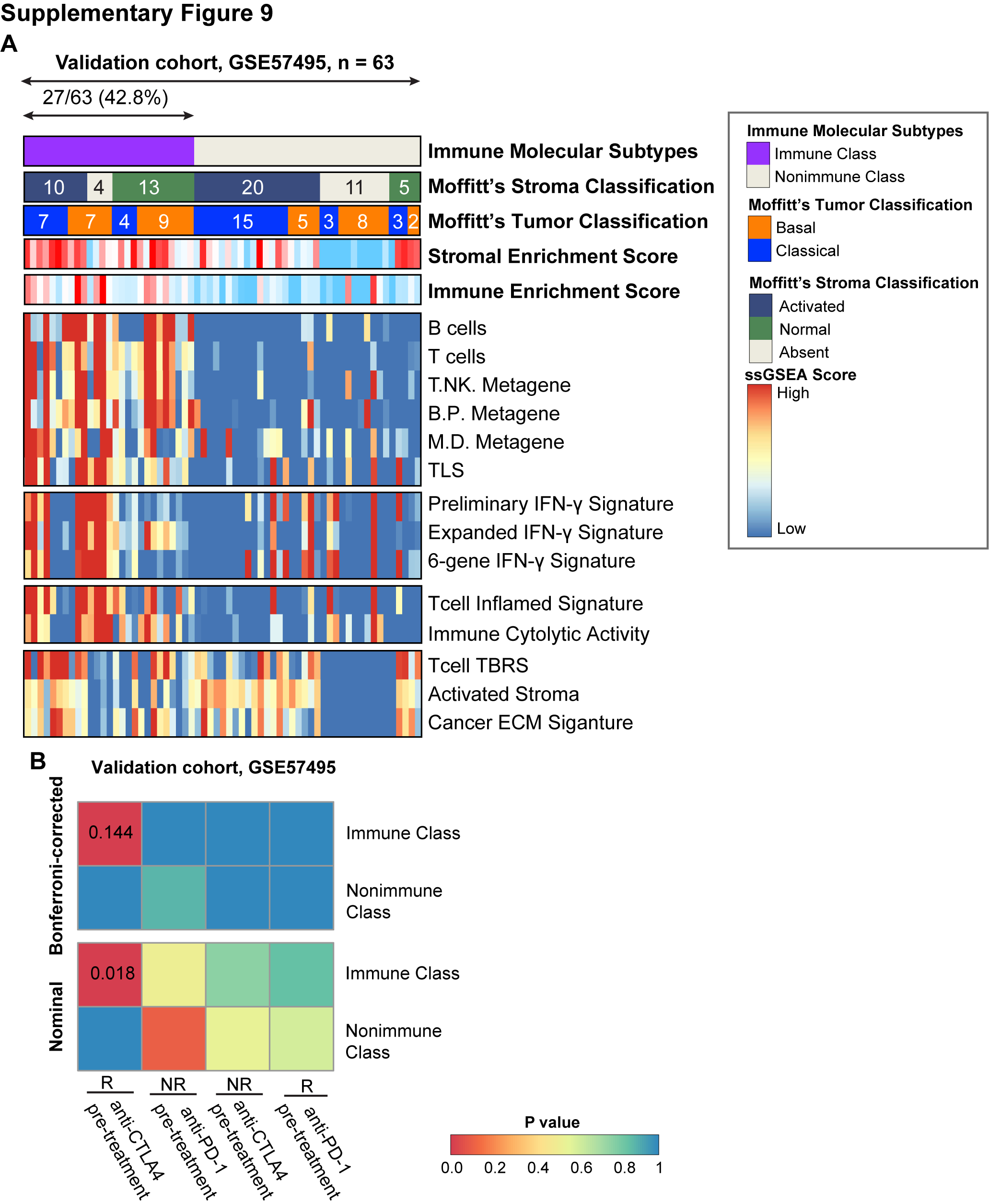

Supplement: Supplementary file 10 [file Image_9.tif]
